# Improving body image at scale among Brazilian adolescents: study protocol for the co-creation and randomised trial evaluation of a chatbot intervention

**DOI:** 10.1186/s12889-021-12129-1

**Published:** 2021-11-20

**Authors:** E. L. Matheson, H. G. Smith, A. C. S. Amaral, J. F. F. Meireles, M. C. Almeida, G. Mora, C. Leon, G. Gertner, N. Ferrario, L. Suarez Battan, J. Linardon, M. Fuller-Tyszkiewicz, P. C. Diedrichs

**Affiliations:** 1 Centre for Appearance Research, Department of Health and Social Sciences, University of the West of England, Coldharbour Lane, Bristol, BS16 1QY UK; 2 Federal Institute of Education, Science and Technology of Southeast of Minas Gerais, 204 Monsenhor José Augusto, Barbacena, 36205018 Brazil; 3 Department of Exercise Science and Sport Management, Kennesaw State University, Kennesaw, GA USA; 4 Department of Psychiatry, Universidade Federal de São Paulo, São Paulo, Brazil; 5 UNICEF Brasília, Office of the Representative of UNICEF in Brazil, SEPN 510, Block A – 2nd floor, Brasília, DF 70750-521 Brazil; 6 Talk2U LLC, 777 Brickell Ave Ste 1210, Miami, Florida 33131 USA; 7 School of Psychology, Deakin University, Geelong, Australia

**Keywords:** Adolescent, Body image, Mental health, Chatbot, Micro-intervention, Brazil, Low- and middle-income countries, Randomised controlled trial, Study protocol

## Abstract

**Background:**

Body image concerns are prevalent among Brazilian adolescents and can lead to poor psychological and physical health. Yet, there is a scarcity of culturally-appropriate, evidence-based interventions that have been evaluated and made widely available. Chatbot technology (i.e., software that mimics written or spoken human speech) offers an innovative method to increase the scalability of mental health interventions for adolescents. The present protocol outlines the co-creation and evaluation of a body image chatbot for Brazilian adolescents via a partnership between academics, industry organisations and the United Nations Children’s Fund (UNICEF).

**Methods:**

A two-armed fully remote randomised controlled trial will evaluate the chatbot’s effectiveness at improving body image and well-being. Adolescent girls and boys (*N* = 2800) aged 13–18 years recruited online will be randomly allocated (1:1) into either: 1) a body image chatbot or 2) an assessment-only control condition. Adolescents will engage with the chatbot over a 72-hour period on *Facebook Messenger*. Primary outcomes will assess the immediate and short-term impact of the chatbot on state- and trait-based body image, respectively. Secondary outcomes will include state- and trait-based affect, trait self-efficacy and treatment adherence.

**Discussion:**

This research is the first to develop an evidence-informed body image chatbot for Brazilian adolescents, with the proposed efficacy trial aiming to provide support for accessible, scalable and cost-effective interventions that address disparities in body image prevalence and readily available resources.

**Trial registration number:**

NCT04825184, registered 30th March 2021.

**Supplementary Information:**

The online version contains supplementary material available at 10.1186/s12889-021-12129-1.

## Background

Despite being an emerging global public mental health issue [1] body image research has largely been confined to high-income, White, English speaking populations [2, 3]. Yet, research from Asia, Africa, and South America highlights cross-cultural similarities in the expression of body image concerns. A study in 26 countries found global normative discontent, with most individuals reporting appearance dissatisfaction [4]. Understanding cross-cultural similarities, as well differences allows for the development of new and/or adaptation of extant evidence-based body image interventions for use in traditionally underserved populations. However, interventions must also be accessible, scalable and cost-effective if they are to achieve impact [5, 6]. Multi-stakeholder partnership is a key mechanism for achieving this. This paper outlines the study protocol for the co-creation and evaluation of a scalable body image chatbot (i.e., software that mimics written or spoken human speech) designed with and for Brazilian adolescents through a partnership between academics, industry organisations (beauty [Dove]; communication and technology [Talk2U]) and the United Nations Children’s Fund (UNICEF).

### Body image in Brazil

With a population of over 212 million people ([7]), body image concerns in Brazil are common across the lifespan, particularly among adolescents. Prevalence of body image concerns range between 26.6–56% and 10.7–36% for adolescent girls and boys, respectively [8–10]. Concurrently, up to 10% of adolescents report engaging in disordered eating and unhealthy weight control behaviours [11, 12]. Further, consumer trends show Brazil is the leading country in diet pill consumption and cosmetic surgery, with 2.5 million surgical and non-surgical aesthetic procedures conducted annually [13]. This is concerning given that body image is a key risk factor for depressive symptoms, smoking, substance misuse and self-harm in other countries [14]. Further, cosmetic procedures incur significant financial expense and have potential medical complications [15]. Together, these findings highlight the urgency for easily-accessible, scalable, and cost-effective interventions designed to address body image concerns among Brazilian populations. At present, such interventions are limited.

Existing body image interventions in Brazil have evolved from international collaborations between Brazilian and US researchers. These include the translation and adaptation of a group-based cognitive dissonance intervention, *The Body Project*, which provides a forum for girls and women to challenge unrealistic appearance ideals and develop body satisfaction. The program proved effective at improving body image, negative affect and resilience to sociocultural pressures, but not disordered eating, among at risk Brazilian adolescent girls [16]. *New Moves*, a US program, was also adapted for use with Brazilian adolescents [17]. This school-based program used a combination of motivational interviewing and exercise and nutrition sessions to improve body dissatisfaction, self-esteem and healthy eating behaviours. Despite its efficacy in improving these outcomes among US girls, it did not lead to significant improvements in these key outcomes among Brazilian girls.

To our knowledge, no evidence-based body image intervention has been developed specifically with and for Brazilian adolescents, nor has a research group utilised technology and multi-disciplinary partnerships to develop, evaluate, disseminate and implement an intervention in Brazil. First, given that 24.3 million young people aged 9–17 years in Brazil (86% of the age range) use the internet [18], digital interventions are likely to be accepted by young people, as well as highly amenable to their lifestyles, which are increasingly digitally oriented. Second, the diverse knowledge and expertise held by the partnership stakeholders ensures that relevant insider knowledge from the respective markets are integrated into the intervention development, evaluation, implementation and dissemination frameworks; thus, making for an innovative and well-considered intervention.

### Digital innovation in body image interventions

The emergence of digital interventions has increased the accessibility, scalability, and cost-effectiveness of mental health interventions [5, 6, 19]. A review into the adaptation of face-to-face body image and eating disorder prevention interventions (e.g., psychoeducation, media literacy and cognitive dissonance) for online use show promising results. However, effects are small and significant issues with attrition and adherence are noted (Cohen’s ds .24–.42) [20, 21]. Consistent with recommendations for using disruptive innovation (e.g., ‘edutainment’; educational content within entertainment settings) and technology to scale up mental health interventions [22], researchers have begun developing and testing purpose-built digital body image interventions. Many of these interventions have been designed to be brief, standalone approaches that provide immediate (‘in-the-moment’) symptom reprieve; an intervention model referred to as ‘micro-interventions’ (e.g., audio and visual clips, online games and e-books) [23]. An advantage of micro-interventions is the ability to embed theoretically driven, evidence-based therapeutic techniques into existing digital platforms and social media networks already frequented by adolescents; thus, potentially removing barriers to dissemination and uptake [5, 6, 22].

Micro-interventions are a promising strategy for addressing mental health among Brazilian adolescents, particularly due to the high percentage of young people with internet access (86%) and a social media network (68% [18];). Recently, micro-interventions have proven effective at producing immediate and short-term body image and mental health benefits among adolescents and young adults in Australia [23], the UK [24] and the US [25]. Until now, chatbot technology has not been developed or evaluated within a micro-intervention framework [26], nor has an evidence-based micro-intervention been purpose-built for Brazilian populations. However, a recent public health initiative by UNICEF Brazil [27], reported high uptake and acceptability of a chatbot addressing sexting among adolescents, with over one million users in the first 12-months of dissemination.

Chatbot technology is a robust, low-barrier alternative to traditional mental health approaches, which are largely dependent on health professionals and services for effective implementation and dissemination [5, 6]. Chatbots are usually hosted on a website or app, with the centralised platform allowing users to engage with a variety of intervention techniques, monitor their symptoms and progress, and at times refer at-risk individuals to more appropriate mental health resources and services [26]. This independence from traditional health care approaches also allows for around the clock access and care, particularly to those who have limited mobility, live distant from health professionals and services, or are reluctant to seek mental health advice due to stigmatization [26]. Therefore, given the prevalence of body image concerns among adolescents, coupled with the scarcity of extant prevention and interventions approaches, as well as adolescent’s frequent online behaviours, chatbot technology offers a new and innovative approach to addressing these concerns at-scale.

### The current trial design

**Fig. 1:**
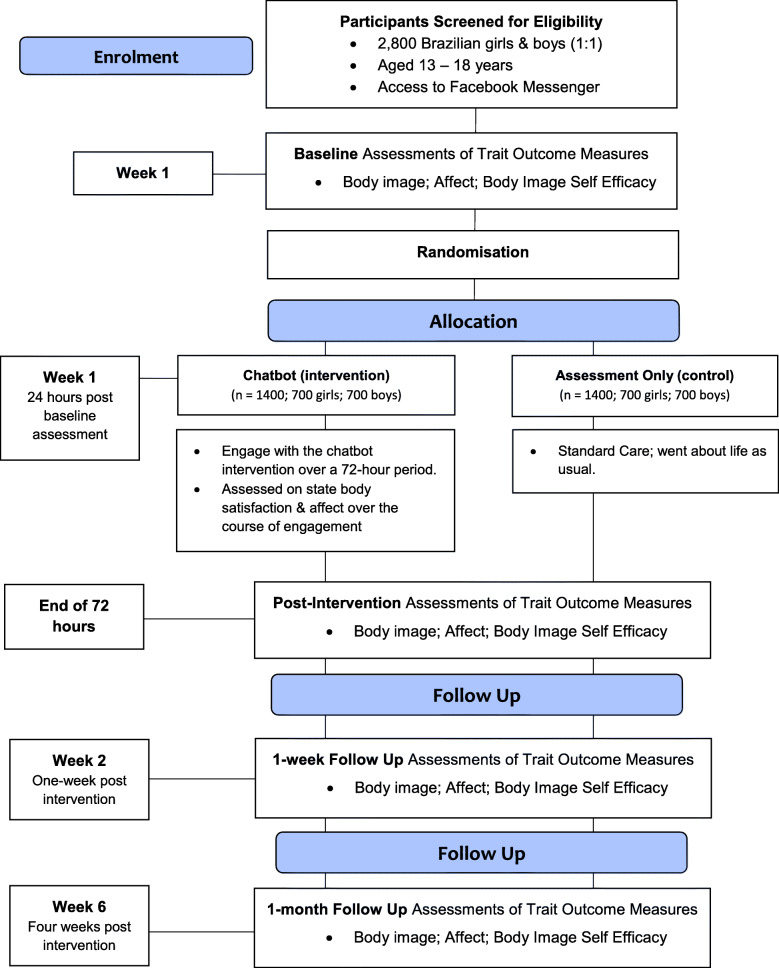
The research design according to the CONSORT EHealth guidelines

**Fig. 2:**
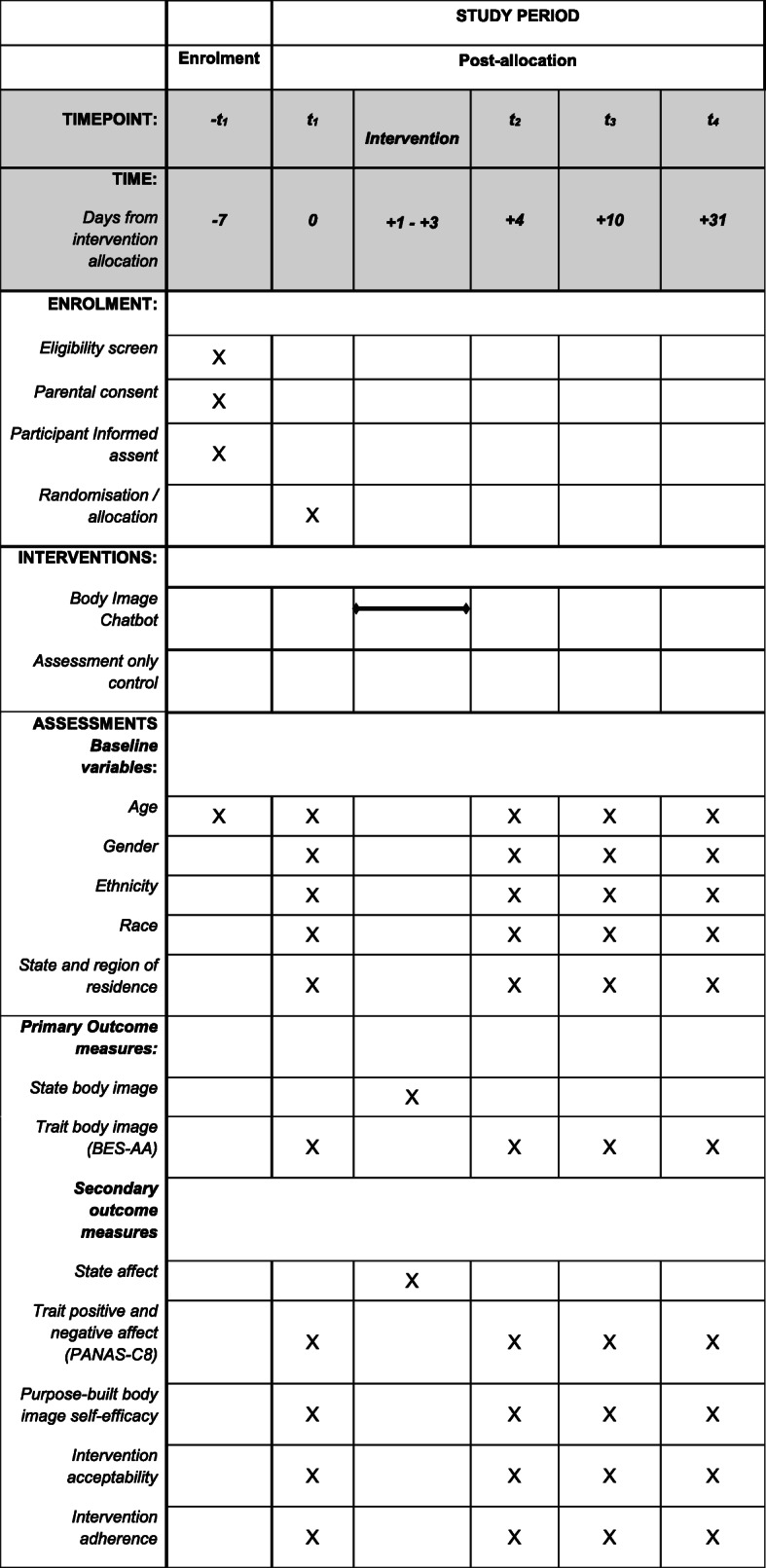
Schedule of enrolment, interventions, and assessments according to the SPIRIT guidelines

This paper outlines the protocol for the co-creation and controlled evaluation of a chatbot targeting risk and protective factors for body image concerns among Brazilian adolescents. A two-arm fully remote randomised controlled trial (Fig. 1) will compare a chatbot intervention to an assessment-only control condition. Participants in the intervention condition will interact with the chatbot over a 72-h period, while participants in the control condition will receive ‘standard care’ for body image concerns among adolescents in Brazil, which at present is no intervention. This protocol paper and the randomised controlled trial design were guided by the Standard Protocol Items: Recommendations for Interventional Trials (SPIRIT; see Fig. 2 and supplementary materials) and the Consolidated Standards of Reporting Trials (CONSORT [28];), including the extension for Electronic Health (CONSORT - EHEALTH [29] see supplementary guidelines).

Assessment of primary and secondary trait outcomes will be conducted at baseline (T1), post-intervention (i.e., 3 days; T2), one-week (T3) and one-month follow-up (T4). Participants in the intervention condition will also be assessed on state-based body satisfaction and affect immediately after engaging with each technique. To our knowledge, this is the first chatbot to target body image among Brazilian adolescents. Therefore, a secondary aim is to assess users’ engagement and adherence. Hypotheses were formulated for overall chatbot efficacy (H1-H2), moderation effects within sub-samples (H3), and intervention engagement and adherence (H4):

- **H1:** The chatbot was designed to provide immediate benefits to users. Therefore, it is anticipated that adolescents will experience improvements in state-based body satisfaction and affect at the time of engagement with the chatbot.
- **H2**: Adolescents randomised into the intervention condition will experience greater improvements in trait-based body esteem, affect and body image self-efficacy at post-intervention, one-week and one-month follow-up, relative to the assessment only control condition.
- **H3:** Based on previous research into the moderating effects of gender [30] and trait psychopathology [31, 32] on body image intervention effectiveness, it was hypothesized that intervention effects will be moderated by gender, and baseline levels of body esteem, affect and body image self-efficacy. Specifically, intervention effects will be greatest among: girls; girls and boys with lower levels of trait body esteem; girls and boys with higher levels of trait negative affect and body image self-efficacy.
- **H4**: With regards to user engagement and adherence, given the novelty of this intervention, analyses will be of an exploratory nature. It is, however, anticipated that greater engagement and adherence with the chatbot intervention will lead to greater improvements in trait-based outcomes.

## Method

### Public and patient involvement

Adolescents were involved in the research since its inception. Adolescents were recruited via UNICEF and provided input on intervention design and development (*N* = 37, 73% girls, age range = 13–19 years), as well as participating in user testing (see Intervention Pilot Study for participant characteristics). Research findings will be disseminated to adolescents and the wider general public via the current industry partner’ and UNICEF’s external communication networks (e.g., social media campaigns).

### Eligibility criteria

The current chatbot is a universal intervention that is designed to provide population-wide benefits, irrespective of an individual’s level of risk. Therefore, increased risk for body image concerns was not an inclusion criterion for this trial. Eligible participants are adolescents aged 13–18 years old; Brazilian Portuguese speaking; Brazilian resident; and, have access to *Facebook Messenger*.

### Intervention

#### Intervention development and content

An evidence-based framework guided the development of the chatbot content, structure and format. This framework allowed for collaborative decision-making processes, whereby scientific theory and research, adolescents’ needs and preferences, and the expertise of project partners were considered throughout [33]. Intervention development phases and the key tasks and decision-making processes associated with these phases are reported in Table 1. These phases resulted in a chatbot, *Topity*, designed for delivery on the *Facebook Messenger* platform. The chatbot targets sociocultural risk and protective factors for body image using eight therapeutic techniques derived from cognitive and behaviour theory for body image [43]. The techniques teach adolescents how to critically analyse and evaluate media content to reduce vulnerability to negative media influences [44]; identify and challenge unhelpful thinking styles and behaviours that perpetuate body image distress [43]; and how to appreciate features and functions of the body beyond appearance [45]. A summary of the intervention topics and the associated change techniques are described in Table 2.

**Table 1:** Intervention development phases and associated tasks

Phase	Key development tasks and decision-making processes
1. Select and appraise the best available scientific research and evidence.	• Critically evaluated extant literature to identify key risk and protective factors for body image among Brazilian adolescents. • Concurrently, UNICEF conducted 26 workshops with 681 (66% girls) ethnically diverse Brazilian adolescents across 15 northern and south-eastern cities to better understand the nature of, and influences on, their body image, and their preferences for the chatbot’s key messages and persona [34].
2. Apply the evidence in a collaborative decision-making process with relevant parties.	• Data from the literature review and focus groups informed a collaborative decision-making process between academics, industry partners and UNICEF to select which risk and protective factors would be targeted within the chatbot. • Three themes were agreed upon: 1) Unrealistic beauty standards perpetuated by society; 2) Appearance pressures experienced within interpersonal relationships; 3) Acceptance and appreciation of one’s body.
3. Selection and adaptation of extant techniques that target the agreed upon themes, for use in the chatbot.	• A micro-intervention framework was used to identify and select therapeutic techniques that addressed the above themes [35]. • Techniques needed to be brief (e.g., completed in under 10 min), standalone (e.g. a distinct beginning and end), immediately actionable (e.g., can be executed in the moment, without additional resources) and adaptable for digital platforms. • Researchers drew on their expertise of working with evidence-based body image prevention and intervention approaches and a meta-analysis on therapeutic change techniques to identify and select appropriate techniques [31]. • Eight techniques (see Table 2) were extracted from extant evidence-based body image programs, including: *Confident Me* [36], *The Body Image Workbook* [37], and *Expand Your Horizon* [38]. • A script was developed for each technique and was reviewed by body image experts from Brazil and the UK, as well as Dove and UNICEF. • Scripts were then amalgamated into a written interactive dialogue to be delivered by the chatbot fictional hosts, Dandara (a young Brazilian woman) and Gabriel (a young Brazilian man). • Hosts’ gender and persona were informed by: ° Mixed-gender preferences observed among young people receiving body image interventions [39]. ° Adolescents’ use of peers for emotional and informational support in online environments ([40]). ° Qualitative and quantitative data obtained by UNICEF via focus groups and an online survey via UReport with girls and boys name preferences.
4. Development of the digital interface	• The digital interface of the chatbot was constructed by communication and technology experts at Talk2U. • This iterative process involved frequent reviews by adolescents, body image experts based in Brazil and the UK, Dove and UNICEF. • Key design features included: °﻿ The use of *Facebook Messenger*, an instant messaging app for smartphones, to host the chatbot. ° The use of interactive elements that enhance user engagement and saliency of key therapeutic messages, including check-in messages, multi-media stimuli (i.e., audio and video clips; emojis [a small digital image or icon used to express an idea or emotion]), virtual hosts and the gamification of intervention techniques. ° In regards to gamification (i.e., the use of game design elements in non-game contexts, [41]), users needed to successfully complete a technique before progressing to the next technique within a thematic cluster. Gamification is used to incentivise users to continue their engagement while allowing them to master easier skills before progressing to more challenging tasks [42]. The ordering of techniques within each thematic cluster was informed by the researchers’ expertise, as well as feedback from further focus groups with adolescents on the acceptability and feasibility of the techniques and presentation formats. • Project partners with technology and marketing expertise advised on the selection of the chatbot’s name, *Topity*. • To reduce bias the institutional and industry affiliations were not displayed on the user interface. Participants and parents were, however, informed of the multi-international partnership and the relevant organisations involved in the ‘Parent and Participant Information & Consent’ form. • Topity can be accessed via Facebook Messenger using the following URL link: https://www.facebook.com/topitychat/

**Table 2:** Chatbot Intervention Topics, Change Techniques and Steps

Topics	Change Technique Objectives	Technique Steps
*Family, friends & body image*		
1. Banish body talk	1. Define and identify forms of body talk. 2. Identify the consequences of body talk and implement strategies for challenging this dialogue.	• User reflects on their own body talk, by estimating the frequency of engagement (e.g., number of times in preceding week), areas of fixation (e.g., belly; legs; skin colour) and the type of commentary (e.g., compliment, criticism, comparison). • Bot led deconstruction task (social media post); user considers the use and frequency of body talk and its impact on the person posting and their followers. • User generates alternative comments that focus on non-appearance aspects in the image (e.g., their affect; the activity, the location); user commits to engaging in this behaviour over the coming week.
2. Dealing with provocative people	1. Identify people that negatively impact your body image. 2. Explore how and when to use assertiveness strategies to address the unhelpful behaviours of others.	• Bot led discussion on how others impact our body image and how these behaviours are framed (e.g., concern, helpful advice, friendly teasing). • User identifies those who negatively impact their body image. • Bot introduces and applies the RIGHTS assertiveness model to an example scenario. • User invited to practice the RIGHTS model with the bot.
*Social media & body image*		
1. The messages behind the media	1. Develop media literacy skills in critically analysing and evaluating media content and the motivations of content creators (e.g., influencers, industry brands). 2. Identify and implement strategies that challenge unrealistic media images and messages, and create safe social media environments.	• Bot led discussion on media literacy; user identifies how media images are manipulated and the motivations of content creators. • Dove Selfie; user watches a 75-second clip on photo editing strategies, indicates their emotive reaction and identifies newly learnt strategies. • Bot describes strategies for creating a realistic, diverse and inclusive social media environment; user commits to engaging in one behaviour in the next week.
2. Unfair-to-compare	1. Define and identify examples of the cognitive distortion, ‘Unfair-to-Compare’. 2. Implement strategies that challenge and reduce upward comparison making.	• Bot led discussion on the internalisation of appearance ideals and upward comparison making. • User reflects on their engagement with this thinking style and the consequences associated with upward comparison making. • Bot describes strategies for challenging the ‘Unfair-to-Compare’ thinking style.
3. A whirlpool of comparisons	1. Identify thoughts and behaviours that contribute to the cycle of comparison making. 2. Identify and implement strategies to stop the cycle from starting or worsening.	• Bot led discussion on the cycle of comparison making; whirlpool analogy. • User identifies their comparison making behaviours and the consequences of these behaviours for themselves and others. • Bot describes strategies for exiting the whirlpool.
4. The magnifying glass	1. Define and identify examples of the cognitive distortion, ‘The Magnifying Glass’. 2. Identify and implement strategies that reduce selective attention on disliked body parts, while increasing attention towards areas that are liked and appreciated.	• Bot led explanation of selective attention and its role in maintaining body image concerns. • User reflects on their areas of fixation (e.g., stomach, legs, body hair). • Bot describes strategies for reducing selective attention. • User invited to engage in a guided mirror exposure task; if unable to in the moment, the audio is saved for a more appropriate time.
*Body appreciation and functionality*		
1. Beauty bound	1. Define and identify examples of the cognitive distortion, ‘Beauty Bound’. 2. Identify and implement strategies that reduce self-imposed body image rules that prevent life engagement.	• Bot led discussion on self-imposed body image rules and limitations; the importance of focusing on the body’s functionality (i.e., what it can do and experience) when attempting to overcome body image rules. • Visualisation activity; user recalls personal example of Beauty Bound thinking (e.g., thoughts, feelings, behaviours and outcome); then visualises the scenario without self-imposed boundaries (e.g., thoughts, feelings, behaviours and outcome); user identifies the differences.
2. Expand your horizon	1. Define body functionality and identify the different forms of functionality. 2. Describe the importance of our own body’s functions.	• Bot led discussion on body functionality (i.e., what the body can do and experience). • Functionality activity; user provided with categories of functionalities and the associated experiences / activities: sensation (i.e., touch, taste, sight); physical activity (e.g., running, cycling, jump-rope), creativity (e.g., cooking, photography, drawing) and self-care (e.g., petting animals, meditation, having a bath). • User selects their most important experience/ activity within each category. • User then reflects on one experience / activity in-depth (e.g., describe what dancing allows the person to experience and why it’s important to them).

#### Intervention pilot study

A pilot study was conducted with 154 Brazilian adolescents to assess *Topity’s* user experience (e.g., ease of use) and acceptability (e.g., enjoyment; content relevance) prior to the main trial. Participants were 13–18-year-old girls and boys (56% girls; *M* = 15.67, SD = 1.62) from geographically diverse regions of Brazil, who were recruited via peer-support leaders, secondary school teachers and non-profit organisation officers known to UNICEF. Following parental consent and participant assent, adolescents were invited to engage with *Topity* as much as they needed over a one-week period. Adolescents assessed the chatbot on 11 acceptability factors, using 7-point response scales (e.g., 1 = *not at all interesting*; 7 = *very interesting*). Factors included: emotive response to the chatbot (e.g., enjoyment, interest, comfortability), relevance (e.g., pertinence, importance and helpfulness of the content to the user and other young people in Brazil), ease of use (e.g., speed and accuracy of responses) and willingness to recommend (e.g., how likely the user would recommend *Topity* to a friend and re-engage in the future). Mean scores for each item, as well as an overall acceptability score are provided in the supplementary materials. Overall, *Topity* was rated highly on overall user experience and acceptability across genders (girls; *M* = 6.40, *SD* = .65; boys; *M* = 6.0, *SD* = 1.03) and age (13–15 years; *M* = 6.41, *SD* = .61; 16–18 years, *M* = 6.07, *SD* = .99). Based on this feedback, no modifications were made to the chatbot prior to the main trial.

### Measures

Research measures are presented in Table 3. One measure was purpose-built for the current research (i.e., body image self-efficacy). Acceptability and adherence measures were informed by existing reviews [52, 53], with remaining measures validated for use among adolescents.

**Table 3:** Research outcomes and internal consistencies

*Demographics*	Age, gender identity, ethnicity, race, state and region of residence	T1
*Primary outcomes*		
State body satisfaction	A single 11-point scale assessing the immediate impact of a chatbot technique on participants’ state body satisfaction (*How satisfied are you with your appearance, right now, in this moment?*). Total score range 0 (*not at all*) to 10 (*very much*). Higher scores reflect greater satisfaction.	Start of chatbot experience. Post-intervention technique. 12-, 16-, 23.5-h after non-engagement.
Trait body satisfaction	Body Esteem Scale for Adolescents & Adults in Brazil [46, 47]. Appearance Positive (AP; e.g., *I like how I look like in photos*), Appearance Negative (AN; e.g., *I feel ashamed about my appearance*) and Weight (e.g., *I am satisfied with my weight*). Mean subscale scores range between 0 (*never*) and 5 (*always*). Higher scores reflect higher esteem. Internal consistency: AP = .85; AN = .88; Weight = .89.	T1- T4
*Secondary outcomes*		
State affect	A single 11-point scale assessing the immediate impact of a chatbot technique on participants’ state affect (*How happy are you, right now, in this moment?*). Total score range between 0 (*not at all*) to 10 (*very much*). Higher scores reflect greater positive affect.	Start of chatbot experience. Post intervention technique. 12-, 16-, 23.5-h after non-engagement.
Trait affect	The Positive and Negative Affect Scale for Children 8-item; PANAS-C8 [48] 8-items related to emotive states (4 positive [e.g., *joyful*]; 4 negative [e.g., *irritated*]). Positive and negative affect subscale scores range between 1 (*not at all*) and 5 (*extremely*). Higher scores reflect greater positive or negative affect. Internal consistency: PA = .77; NA = .76	T1 - T4
Trait body image self- efficacy	A purpose-built measure, The Body Image Self-Efficacy Scale, derived from existing self-efficacy measures [49–51]. 5 visual analogue scales (VAS; 0 [*not at all confiden*t] to 100 [*very confident*]) assessing participants’ belief in their ability to execute strategies to improve their body image (e.g., *I am able to learn and practice new skills to help improve my body image*). Total mean scores range between 0 and 100. Higher scores reflect greater body image self-efficacy. Internal consistency: .82	T1 - T4
Intervention adherence	Digital metrics will assess participants engagement with the chatbot, including: • Percentage of participants who complete the intervention (e.g., minimum of 1 technique completed over the 72-h intervention period); • Percentage of participants who enter the intervention but do not complete the intervention; • Percentage of participants who complete each intervention technique; • Average number of techniques completed; • Average length of time taken to complete each technique; • Average length of time spent engaging with the chatbot over the 72-h period.	72-Hour Intervention Period
Intervention acceptability	11 items assessed participants acceptability of the chatbot. Factors included: • Emotive response to the chatbot (e.g., enjoyment, interest, comfortability); • Relevance (e.g., pertinence, importance and helpfulness of the content to the user and other young people in Brazil); • Ease of use (e.g., speed and accuracy of responses); and • Willingness to recommend (e.g., how likely the user would recommend Topity to a friend and re-engage in the future). Item scores range between 1 (e.g., *not at all enjoyable*) and 7 (e.g., *very enjoyable*). Higher scores reflect greater acceptability.	T4

Note. T1 = Baseline; T1 = Baseline; T2 = Post-intervention; T3 = One-Week Follow-Up; T4 = One-Month Follow-up

### Participant recruitment and procedure

A community sample of 2800 adolescents aged 13–18 from diverse ethnic, geographic and socio-economic backgrounds will be recruited via a Brazilian research agency’s recruitment panels (i.e., via email to their participant databases) and via UNICEF’s online communication platforms (e.g., U-Report^1^). Recruitment will occur 2 weeks prior to the trial commencing. Given UNICEF’s reach and expertise with underserved adolescents in Brazil, the inclusion of this recruitment channel will ensure that a diverse sample is approached for participation. Further, recruitment numbers across these metrics (e.g., geography) will be regularly monitored and addressed by the research team to ensure diversity across the sample is achieved. Recruitment materials will include a link to an online screener questionnaire to determine participant eligibility. After being screened as eligible, participants will be emailed an information pack to be read with their parent or guardian. Parents will provide consent as per the Brazilian General Data Protection Law, by uploading their signature to online identification verification software that is monitored by the research agency. Following parental consent, adolescents will complete the online self-report assessment (T1), which will commence with adolescents providing informed assent. Those who do no assent will not proceed with the trial. On completion of the survey, adolescents will be randomised into one of two conditions: intervention or assessment-only. Those in the intervention condition will receive a link to the chatbot 24-hours after completing the baseline assessment. Those in the assessment-only condition will be provided with a link to the chatbot after they have completed all assessment timepoints.

Adolescents in the intervention condition will be encouraged to engage with the chatbot as much as possible over a 72-hour period. During this time, participants may complete the eight intervention techniques as many times as they like. Participants will be assessed on state body satisfaction and affect when they first enter the chatbot and immediately after engaging with a technique. If participants do not engage with the chatbot after 12-, 16 and 23.5 hours, they will receive a prompting notification that encourages engagement with the chatbot. That is, if they do not engage after the 12-hour prompt, they will receive up to two more prompts.

At completion of the 72-hour intervention period, both conditions will be assessed on primary and secondary trait outcomes immediately post-intervention and, at one-week and one-month follow-up. Those in the intervention condition will be encouraged to continue using the chatbot. Following the one-month follow-up assessment, all participants will be debriefed on study aims and objectives, and provided with mental health resources accessible in Brazil. Assessment-only participants will be invited to engage with the chatbot; however, their engagement will not be monitored or assessed. Lastly, to compensate participants for their data usage, they will receive an electronic voucher to the value of R$100 and R$80, for the intervention and control condition, respectively. Participants were not notified of the compensation value until study completion. 

### Randomisation and blinding

In the present trial, adolescents will be randomised to one of two conditions based on a permuted block design, using survey software by the research agency. Randomisation will be performed on an individual level to ensure timely allocation, while maintaining a balance of participants across conditions. Participants cannot be blinded to allocation due to the behavioural nature of the two conditions; however, they will be blinded to the study aims and hypotheses. As randomisation is conducted by an external research agency, researchers will be blinded to participant allocation. Data analysts will be blinded to condition allocation (dummy coded) when conducting statistical analyses on primary and secondary trait outcomes; however, blind analyses cannot be conducted on primary and secondary state measures due to the within-group design. State and trait data will be separated into two data files to ensure blinding is maintained for trait-based measures.

### Statistical power

The limited number of prior micro-interventions and insufficient detail for key parameters complicates efforts to confidently generate all required parameter estimates for power calculations. Accordingly, we adopted a Monte Carlo simulation approach that tested power across a range of combinations of key statistical parameters for multilevel modelling: within-person sample size (varying from 3 to 5 estimates per person), intraclass correlation (ICC) for outcome (ranging from .3 to .6 in recognition that ICC values tend to be large for repeated measures designs), random slope variance (choosing .01, .09, and .25 to reflect small, moderate, and large amounts of variance [54]), and effect size (setting at *r* = .2 to reflect small yet practically meaningful effect size [55]).

Using the SIMR (simulation-based power calculations for mixed models) package in R [56], we tested power across these different combinations of parameter estimates, with power set at .80, alpha = .05 (two-tailed), equal allocation to intervention and control arms, and conservatively allowing for 25% attrition on an initial sample of 2800 participants at the final timepoint. We note too that Hypotheses 1 and 4 are limited to the intervention arm, and thus the sample size used for those calculations was 1050 (1400 participants with 25% attrition). Using these inputs, our simulations indicated power was > .99 for all cross-level interaction effects regardless of input values, and > .80 for all within-person effects with the exception of scenarios where: (1) the random slope variance was small, or (2) a small-to-moderate random slope variance was accompanied by a small number of within-person estimates (average cluster size of 3–4). These scenarios of small random variance coupled with a small estimated effect size are unlikely based on best available evidence from past studies [23–25]. Thus, our sample size is likely to be sufficient across a range of plausible data scenarios.

### Data analyses

At trial completion, Toluna and Talk2U will deliver password protected datafiles to the corresponding author who will clean, screen and separate state- and trait-based data for blinding purposes. Data will then be delivered to the data analysts. Analyses will be conducted in Stata. A series of linear mixed effects models will test hypotheses. H1 analyses will be limited to intervention arm participants as there is no comparable pre- and post-technique data for the assessment-only arm, while analyses for H2, H3 and H4 will include participants from both study arms.

To test H1, the immediate effects of intervention content on state-based outcomes will be explored by regressing state-based outcome measures (body satisfaction and affect) onto a time variable (0 = pre-content, 1 = post-content). For H2-H4, scores on trait-level outcome variables will be regressed onto a dummy coded time variable, with baseline as the reference category compared against post-intervention, one-week, and one-month follow-up timepoints, using random effects to allow for individual differences in amount of change in symptoms over time. Group (control = 0, intervention = 1) will be included as a cross-level moderator to test group differences in change over time (H2). The moderating effect of baseline variables on this group effect will be conducted to test H3. In evaluating H4, adherence and engagement measures will be tested as moderators of change in trait-based outcome measures over time (relative to baseline) for intervention participants. Relations among random effects of Level 1 time variables will be modelled with an unstructured covariance matrix.

Baseline differences between intervention and control participants in terms of demographic factors will be tested. Any variables shown to differ across groups will be included as covariates in analyses. Linear mixed model results will be presented in adjusted and unadjusted forms to demonstrate impact of these covariates on results.

Analyses will be conducted on an intention-to-treat basis, with participant data included based on group assignment at baseline. As the post-technique state-based measure is an indicator of use of the intervention resources, only instances where participants provide data for both pre- and post-technique will be included in analyses for H1. Missing data for group comparisons (H2) and baseline moderation effects (H3) will be handled using multiple imputation by chained equations with 50 imputations. Pattern mixture modelling using the mimix package [57] will be applied to determine sensitivity of results to non-ignorable missingness.

### Ethical issues, approval and trial registration

The research will be conducted in accordance with the ethical standards and guidelines for conducting research with young people in Brazil. Study participation is voluntary. Prior to consent and assent, parents and participants will be provided with an information sheet outlining the research procedures and associated risks. Consent withdrawal is possible at any time without cause for justification. At study completion participants will be provided with a debrief form disclosing research aims and ancillary mental health sources.

The associated ethics committees will be informed of any severe adverse events or other unintended effects of the trial intervention. This study received ethics approval from the Instituto Federal de Educação, Ciência e Tecnologia do Sudeste de Minas Gerais (4.232.804), Comissão Nacional de Ética em Pesquisa (4.582.466), and the University of the West of England (HAS 19.12.090). The study is registered with Clinical Trials. Gov (NCT04825184; website: https://clinicaltrials.gov/show/NCT04825184).

### Dissemination

Research findings will be disseminated via open-access peer reviewed publications and academic conferences, as well as beyond the scientific community. Findings will be shared through infographics and videos to interested members of the public, including on the social media platforms and websites of the Dove Self-Esteem Program, UNICEF and Talk2U. If the intervention proves effective, the chatbot will be scaled up for delivery to adolescents across Brazil via social media marketing campaigns targeting audiences directly on *Facebook*, *Instagram* and *TikTok*.

## Discussion

The current study will be the first to develop and test a chatbot that addresses risk and protective factors for body image, as well as the first to design - rather than adapt - a body image intervention specifically for Brazilian adolescents. The two-armed fully remote randomised controlled trial will assess the chatbot’s efficacy at eliciting immediate and short-term improvements in adolescents’ state- and trait-based body image, affect and self-efficacy, as well as users’ intervention engagement and adherence. At the completion of the trial, provided the chatbot is efficacious and does not require major revisions (e.g., is found ineffective or harmful), the chatbot will be made freely available to Brazilian adolescents in the latter part of 2021.

### Strengths and future impact

The current research makes several advancements in addressing mental health demands of young people at-scale and builds upon previous work in innovative ways. First, our international multi-disciplinary partnership between academics, industry organisations and UNICEF provide the expertise and infrastructure to develop, evaluate and disseminate a highly scalable evidence-based intervention for adolescents. Second, collaborative decision-making processes between adolescents and project partners, as well as a small internal pilot ensured the chatbot was highly acceptable among the target audience; a key moderator of intervention efficacy [52]. Third, the chatbot is hosted on *Facebook Messenger*, a free and widely available online platform. Hence, intervention implementation and dissemination are independent from health professionals, services and physical locations (e.g., schools, clinics), and thus reduce barriers to health care and increase engagement among hard-to-reach populations [26]. Relatedly, a micro-intervention framework was used to develop and evaluate the chatbot; an intervention model yet to be utilised among Brazilian adolescents. Findings from this research will further our understanding of micro-intervention effectiveness at providing immediate and short-term mental health reprieve. Lastly, body image concerns are a global issue among adolescents. Therefore, if efficacious, the chatbot may be translated and adapted for use among adolescents in other cultures and countries; thus, further expanding the reach and impact of the current research.

### Limitations

The current study will not be without limitations, including the use of a fully remote, online randomised controlled trial design and an assessment-only control condition. Reviews into online interventions indicate that this intervention modality is associated with low adherence and high attrition rates in trials [20, 26]. These factors were considered during intervention development and research design phases. Intervention features such as check-in messages, multi-media stimuli (i.e., audio and video clips; emojis [a small digital image or icon used to express an idea or emotion]), virtual hosts and the gamification of intervention techniques were incorporated to enhance user engagement, experience and acceptability. Further, the study is sufficiently powered to detect effects within intention-to-treat and completer samples. Recruitment and data collection will be outsourced to an internationally recognised research agency who specialise in large-scale data collection; thus, ensuring the randomised controlled trial is conducted in a timely and effective manner with a nationally representative sample. Lastly, an assessment-only control condition was informed by a public health framework for assessing interventions in real world settings, and comparing them to ‘care as usual’ [58]. Given the scarcity of body image interventions for adolescents, receipt of no intervention was deemed appropriate.

## Conclusion

The current research addresses an unmet need for scalable evidence-based body image resources for adolescents. This protocol paper describes the collaborative process used by an international multi-disciplinary partnership to develop an evidence-based body image chatbot for use among Brazilian adolescents. Further, the paper provides a detailed overview of a planned randomised controlled trial, assessing the chatbot’s efficacy on eliciting immediate and short-term improvements in adolescents’ body image and related attitudes. If proven effective, the chatbot has the potential to address adolescents’ body image concerns at-scale and therefore reduce the disparities between health-care demands and supply.

## Supplementary Information

**Additional file 1.**

## Data Availability

The body image chatbot, *Topity*, will be freely available to those with access to *Facebook Messenger*. Anonymised survey data will be available on reasonable request for non-commercial purposes.

## Abbreviations

DSEP: Dove self-esteem project
ICC: Intraclass correlations
H1, H2, H3, H4: Hypothesis one, hypothesis two, hypothesis three, hypothesis four
SIMR: Simulation-based power calculations for mixed models
T1, T2, T3: Time one, time two, time three
UNICEF: United Nations Children’s Fund

## Footnotes

1 U-Report is a free tool for community participation, designed to address issues that the population cares about. https://ureport.in/about/
