# Supplementary material for: Improving body image at scale among Brazilian adolescents: study protocol for the co-creation and randomised trial evaluation of a chatbot intervention
Source: BMC Public Health. 2021 Nov 20;21:2135. doi: 10.1186/s12889-021-12129-1 (PMC8605542; doi:10.1186/s12889-021-12129-1)
Supplement: Supplementary file 1 — Additional file 1. [file 12889_2021_12129_MOESM1_ESM.docx]

Supplementary Materials

Supplementary Table 1

SPIRIT 2013 Checklist: Recommended items to address in a clinical trial protocol and related documents

| **Section/item** | **Item No** | **Description** | **Page Ref** |
| --- | --- | --- | --- |
| **Administrative information** | | | |
| Title | 1 | Descriptive title identifying the study design, population, interventions, and, if applicable, trial acronym | 1 |
| Trial registration | 2a | Trial identifier and registry name. If not yet registered, name of intended registry | 2, 15 |
|  | 2b | All items from the World Health Organization Trial Registration Data Set | N/A |
| Protocol version | 3 | Date and version identifier | 1 |
| Funding | 4 | Sources and types of financial, material, and other support | 19 |
| Roles and responsibilities | 5a | Names, affiliations, and roles of protocol contributors | 1, 19 |
|  | 5b | Name and contact information for the trial sponsor | 1 |
|  | 5c | Role of study sponsor and funders, if any, in study design; collection, management, analysis, and interpretation of data; writing of the report; and the decision to submit the report for publication, including whether they will have ultimate authority over any of these activities | 19, 20 |
|  | 5d | Composition, roles, and responsibilities of the coordinating centre, steering committee, endpoint adjudication committee, data management team, and other individuals or groups overseeing the trial, if applicable (see Item 21a for data monitoring committee) | N/A |

| **Introduction** | | | |
| --- | --- | --- | --- |
| Background and rationale | 6a | Description of research question and justification for undertaking the trial, including summary of relevant studies (published and unpublished) examining benefits and harms for each intervention | 3-7 |
|  | 6b | Explanation for choice of comparators | 6 |
| Objectives | 7 | Specific objectives or hypotheses | 7 |
| Trial design | 8 | Description of trial design including type of trial (e.g., parallel group, crossover, factorial, single group), allocation ratio, and framework (e.g., superiority, equivalence, noninferiority, exploratory) | 6-7, 30 |
| **Methods: Participants, interventions, and outcomes** | | | |
| Study setting | 9 | Description of study settings (e.g., community clinic, academic hospital) and list of countries where data will be collected. Reference to where list of study sites can be obtained | 10-12 |
| Eligibility criteria | 10 | Inclusion and exclusion criteria for participants. If applicable, eligibility criteria for study centres and individuals who will perform the interventions (e.g., surgeons, psychotherapists) | 8 |
| Interventions | 11a | Interventions for each group with sufficient detail to allow replication, including how and when they will be administered | 8, 33-38 |
|  | 11b | Criteria for discontinuing or modifying allocated interventions for a given trial participant (e.g., drug dose change in response to harms, participant request, or improving/worsening disease) | N/A |
|  | 11c | Strategies to improve adherence to intervention protocols, and any procedures for monitoring adherence (e.g., drug tablet return, laboratory tests) | 10-12, 17, 33-38 |
|  | 11d | Relevant concomitant care and interventions that are permitted or prohibited during the trial | N/A |
| Outcomes | 12 | Primary, secondary, and other outcomes, including the specific measurement variable (e.g., systolic blood pressure), analysis metric (e.g., change from baseline, final value, time to event), method of aggregation (e.g., median, proportion), and time point for each outcome. Explanation of the clinical relevance of chosen efficacy and harm outcomes is strongly recommended | 10, 39-41 |
| Participant timeline | 13 | Time schedule of enrolment, interventions (including any run-ins and washouts), assessments, and visits for participants. A schematic diagram is highly recommended (see Figure) | 30-32 |
| Sample size | 14 | Estimated number of participants needed to achieve study objectives and how it was determined, including clinical and statistical assumptions supporting any sample size calculations | 12-13 |
| Recruitment | 15 | Strategies for achieving adequate participant enrolment to reach target sample size | 10-11 |
| **Methods: Assignment of interventions (for controlled trials)** | | | |
| Allocation: |  |  |  |
| Sequence generation | 16a | Method of generating the allocation sequence (e.g., computer-generated random numbers), and list of any factors for stratification. To reduce predictability of a random sequence, details of any planned restriction (e.g., blocking) should be provided in a separate document that is unavailable to those who enrol participants or assign interventions | 12 |
| Allocation concealment mechanism | 16b | Mechanism of implementing the allocation sequence (e.g., central telephone; sequentially numbered, opaque, sealed envelopes), describing any steps to conceal the sequence until interventions are assigned | 12 |
| Implementation | 16c | Who will generate the allocation sequence, who will enrol participants, and who will assign participants to interventions | 12 |
| Blinding (masking) | 17a | Who will be blinded after assignment to interventions (e.g., trial participants, care providers, outcome assessors, data analysts), and how | 12 |
|  | 17b | If blinded, circumstances under which unblinding is permissible, and procedure for revealing a participant’s allocated intervention during the trial | 12 |
| **Methods: Data collection, management, and analysis** | | | |
| Data collection methods | 18a | Plans for assessment and collection of outcome, baseline, and other trial data, including any related processes to promote data quality (e.g., duplicate measurements, training of assessors) and a description of study instruments (e.g., questionnaires, laboratory tests) along with their reliability and validity, if known. Reference to where data collection forms can be found, if not in the protocol | 10-12, 39-41 |
|  | 18b | Plans to promote participant retention and complete follow-up, including list of any outcome data to be collected for participants who discontinue or deviate from intervention protocols | 10-12, 17 |
| Data management | 19 | Plans for data entry, coding, security, and storage, including any related processes to promote data quality (e.g., double data entry; range checks for data values). Reference to where details of data management procedures can be found, if not in the protocol | 12, 13 |
| Statistical methods | 20a | Statistical methods for analysing primary and secondary outcomes. Reference to where other details of the statistical analysis plan can be found, if not in the protocol | 13-15 |
|  | 20b | Methods for any additional analyses (e.g., subgroup and adjusted analyses) | 13-14 |
|  | 20c | Definition of analysis population relating to protocol non-adherence (e.g., as randomised analysis), and any statistical methods to handle missing data (e.g., multiple imputation) | 13-14 |
| **Methods: Monitoring** | | | |
| Data monitoring | 21a | Composition of data monitoring committee (DMC); summary of its role and reporting structure; statement of whether it is independent from the sponsor and competing interests; and reference to where further details about its charter can be found, if not in the protocol. Alternatively, an explanation of why a DMC is not needed | Not included in the manuscript |
|  | 21b | Description of any interim analyses and stopping guidelines, including who will have access to these interim results and make the final decision to terminate the trial | N/A |
| Harms | 22 | Plans for collecting, assessing, reporting, and managing solicited and spontaneously reported adverse events and other unintended effects of trial interventions or trial conduct | 15 |
| Auditing | 23 | Frequency and procedures for auditing trial conduct, if any, and whether the process will be independent from investigators and the sponsor | Not included in the manuscript |
| **Ethics and dissemination** | | | |
| Research ethics approval | 24 | Plans for seeking research ethics committee/institutional review board (REC/IRB) approval | 15,18 |
| Protocol amendments | 25 | Plans for communicating important protocol modifications (e.g., changes to eligibility criteria, outcomes, analyses) to relevant parties (e.g., investigators, REC/IRBs, trial participants, trial registries, journals, regulators) | 15 |
| Consent or assent | 26a | Who will obtain informed consent or assent from potential trial participants or authorised surrogates, and how (see Item 32) | 10-11 |
|  | 26b | Additional consent provisions for collection and use of participant data and biological specimens in ancillary studies, if applicable | N/A |
| Confidentiality | 27 | How personal information about potential and enrolled participants will be collected, shared, and maintained in order to protect confidentiality before, during, and after the trial | 10-12 |
| Declaration of interests | 28 | Financial and other competing interests for principal investigators for the overall trial and each study site | 19 |
| Access to data | 29 | Statement of who will have access to the final trial dataset, and disclosure of contractual agreements that limit such access for investigators | 18 |
| Ancillary and post-trial care | 30 | Provisions, if any, for ancillary and post-trial care, and for compensation to those who suffer harm from trial participation | 12, 15 |
| Dissemination policy | 31a | Plans for investigators and sponsor to communicate trial results to participants, healthcare professionals, the public, and other relevant groups (e.g., via publication, reporting in results databases, or other data sharing arrangements), including any publication restrictions | 15,19 |
|  | 31b | Authorship eligibility guidelines and any intended use of professional writers | 19 |
|  | 31c | Plans, if any, for granting public access to the full protocol, participant-level dataset, and statistical code | 18 |
| **Appendices** | | | |
| Informed consent materials | 32 | Model consent form and other related documentation given to participants and authorised surrogates | Supplementary  materials |
| Biological specimens | 33 | Plans for collection, laboratory evaluation, and storage of biological specimens for genetic or molecular analysis in the current trial and for future use in ancillary studies, if applicable | N/A |

Supplementary Table 2

CONSORT 2010 Checklist and Extension Items for Randomised Trials Including E-Health

| Section | CONSORT 2010 Item | | | | | | Addressed on Page No | | CONSORT-EHEALTH Extension Item | | | | Addressed on Page No |  |
| --- | --- | --- | --- | --- | --- | --- | --- | --- | --- | --- | --- | --- | --- | --- |
| **Title and Abstract** | | | | | | | | | | | | | |  |
| **Title and Abstract** | 1a | | | Identification as a randomised trial in the title | | | 1 | | CONSORT-EHealth 1a, b | 1a (i) Identify the mode of delivery in the title | | | 1 |  |
|  |  |  |  |  |  |  |  |  |  | 1a (ii) Mention non-web-based components or important co-interventions in the title | | | N/A |  |
|  |  |  |  |  |  |  |  |  |  | 1a (iii) Mention primary condition or target group in the title | | | 1 |  |
|  |  |  |  |  |  |  |  |  |  | 1b (i) Mention key features/functionalities/components of the intervention and comparator in the abstract | | | 2 |  |
|  | 1b | | | Structured summary of trial design, methods, results, and conclusions (for specific guidance see CONSORT for abstracts) | | | 2 | |  | 1b (ii) Clarify the level of human involvement in the abstract | | | 2 |  |
|  |  |  |  |  |  |  |  |  |  | 1b (iii) Open vs. closed, web-based (self-assessment) vs. face-to-face assessments in abstract | | | 2 |  |
|  |  |  |  |  |  |  |  |  |  | 1b (iv) Results in abstract must contain use data | | | N/A |  |
|  |  |  |  |  |  |  |  |  |  | 1b (v) Conclusions/Discussions in abstract for negative trials | | | N/A |  |
| **Introduction** | | | | | | | | | | | | | |  |
| **Background and objectives** | 2a | | | | Scientific background and explanation of rationale | | 3-7 | | CONSORT-EHealth 2a, b | 2a (i) Describe the problem and the type of system/solution that is object of the study | | 3-7 | |  |
|  |  |  |  |  |  |  |  |  |  | 2a (ii) Scientific background, rationale: What is known about the type of system that is the object of the study. Briefly justify the choice of the comparator | | 3-7 | |  |
|  | 2b | | | | Specific objectives or hypotheses | | 7 | |  | **2b: No EHealth-specific additions** | |  | |  |
| **Method** | | | | | | | | | | | | | |  |
| **Trial design** | 3a | | | | Description of trial design (such as parallel, factorial) including allocation ratio | | 6-7 | | CONSORT -EHealth 3a, b | **3a: No EHealth-specific additions** |  | | |  |
|  | 3b | | | | Important changes to methods after trial commencement (such as eligibility criteria), with reasons | | N/A | |  | 3b (i) Bug fixes, Downtimes, Content Changes: A description of changes to methods, including important changes made on the intervention or comparator during the trial. | N/A | | |  |
| **Participants** | 4a | | | | Eligibility criteria for participants | | 8, 10-11 | | CONSORT – EHealth 4a, b | 4a (i) Computer / Internet literacy | 8, 10-11 | | |  |
|  |  |  |  |  |  |  |  |  |  | 4a (ii) Open vs. closed, web-based vs. face-to-face assessments | 10-11 | | |  |
|  |  |  |  |  |  |  |  |  |  | 4a (iii) Information given during recruitment | 10-11 | | |  |
|  | 4b | | | | Settings and locations where the data were collected | | 8, 10-11 | |  | 4b (i) Clearly report if outcomes were (self-) assessed through online questionnaires | 10-11 | | |  |
|  |  |  |  |  |  |  |  |  |  | 4b (ii) Report how institutional affiliations are displayed | 35 | | |  |
| **Interventions** | 5 | | | | The interventions for each group with sufficient details to allow replication, including how and when they were actually administered | | 8-9; 33-35 | | CONSORT – EHealth 5 | 5 (i) Mention names, credential, affiliations of the developers, sponsors, and owner | 19-20 | | |  |
|  |  |  |  |  |  |  |  |  |  | 5 (ii) Describe the history/development process | 33-35 | | |  |
|  |  |  |  |  |  |  |  |  |  | 5 (iii) Revisions and updating: Clearly mention the date and/or version number of the application/intervention | - | | |  |
|  |  |  |  |  |  |  |  |  |  | 5 (iv) Provide information on quality assurance methods to ensure accuracy and quality of information provided | - | | |  |
|  |  |  |  |  |  |  |  |  |  | 5 (v) Ensure replicability by publishing the source code, and/or providing screenshots/screen-capture video, and/or providing flowcharts of the algorithms used | Supplementary Materials | | |  |
|  |  |  |  |  |  |  |  |  |  | 5 (vi) Digital preservation: Provide the URL of the application. | 35 | | |  |
|  |  |  |  |  |  |  |  |  |  | 5 (vii) Access: Describe how participants accessed the application and in what setting/context. | 8, 10-11 | | |  |
|  |  |  |  |  |  |  |  |  |  | 5 (viii) Describe mode of delivery, features/functionalities/components of  the intervention and comparator, and the theoretical framework used to design them (instructional strategy behavior change  techniques, persuasive features). | 8-9; 33-35 | | |  |
|  |  |  |  |  |  |  |  |  |  | 5 (ix) Describe use parameters: Clarify what instructions or recommendations were given to the user (e.g., regarding timing, frequency, heaviness of use). | 8-11 | | |  |
|  |  |  |  |  |  |  |  |  |  | 5 (x) Clarify the level of human involvement | 6 | | |  |
|  |  |  |  |  |  |  |  |  |  | 5 (xi) Report any prompts/reminders used: Clarify if there were prompts to use the application, what triggered them, frequency. | 11 | | |  |
|  |  |  |  |  |  |  |  |  |  | 5 (xii) Describe any co-interventions: Clearly state any “interventions that are provided in addition to the targeted eHealth intervention” | N/A | | |  |
| **Outcomes** | 6a | | | | Completely defined pre-specified primary and secondary outcome measures, including how and when they were assessed | | 11-13; 30-32; 39-41 | | CONSORT – EHealth 6a, b | 6a (i) If outcomes were obtained through online questionnaires, describe if they were validated for online use [6] and apply CHERRIES items to  describe how the questionnaires were designed/deployed | 10 | | |  |
|  |  |  |  |  |  |  |  |  |  | 6a (ii) Describe whether and how “use” was defined, measured and/ monitored. Use/adoption metrics are important process outcomes that should be reported in any ehealth trial. | 41 | | |  |
|  |  |  |  |  |  |  |  |  |  | 6a (iii) Describe whether, how, and when qualitative feedback was obtained from participants. | 8, 33-35 | | |  |
|  | 6b | | | | Any changes to trial outcomes after the trial commenced, with reasons | | N/A | |  | **6b: No EHealth-specific additions** |  | | |  |
| **Sample size** | 7a | | | | How sample size was determined | | 12-13 | | CONSORT – EHealth 7a, b | 7a (i) Describe whether and how expected attrition was considered when calculating the sample size. | 12-13 | | |  |
|  | 7b | | | | When applicable, explanation of any interim analyses and stopping guidelines | | N/A | |  | **7b: No EHealth-specific additions** |  | | |  |
| **Randomisation** | | | | | | | | | | | | | |  |
| **Sequence generation** | 8a | | | | Method used to generate the random allocation sequence | | | 12 | CONSORT – EHealth 8a, b | **8a, b: No EHealth-specific additions** |  | | |  |
|  | 8b | | | | Type of randomisation; details of any restriction (such as blocking and block size) | | | 12 |  |  |  |  |  |  |
| **Allocation concealment mechanism** | 9 | | | | Mechanism used to implement the random allocation sequence (such as sequentially numbered containers), describing any steps taken to conceal the sequence until interventions were assigned | | | 12 | CONSORT – EHealth 9 | **9: No EHealth-specific additions** |  | | |  |
| **Implementation** | 10 | | | | Who generated the random allocation sequence, who enrolled participants, and who assigned participants to interventions | | | 12 | CONSORT – EHealth 10 | **10: No EHealth-specific additions** |  | | |  |
| **Blinding** | 11a | | | | If done, who was blinded after assignment to interventions (for example, participants, care providers, those assessing outcomes) and how | | | 12 | CONSORT – EHealth 11a, b | 11a (i) Specify who was blinded, and who wasn’t | 12 | | |  |
|  |  |  |  |  |  |  |  |  |  | 11a (ii) Informed consent procedures (4a-ii) can create biases and certain expectations - discuss whether participants knew which intervention was the “intervention of interest” and which one was the “comparator. | 12 | | |  |
|  | 11b | | | | If relevant, description of the similarity of interventions | | | N/A |  | **11b: No EHealth-specific additions** |  | | |  |
| **Statistical methods** | 12a | | | | Statistical methods used to compare groups for primary and secondary outcomes | | | 13-14 | CONSORT – EHealth 12a, b | 12a (i) Imputation techniques to deal with attrition / missing values | 13-14 | | |  |
|  | 12b | | | | Methods for additional analyses, such as subgroup analyses and adjusted analyses | | | 13-14 |  | **12b: No EHealth-specific additions** |  | | |  |
| **Results** | | | | | | | | | | | | | |  |
| **Participant flow** (a diagram is strongly recommended) | | | 13a | | For each group, the numbers of participants who were randomly assigned, received intended treatment, and were analysed for the primary outcome | | | 31 | CONSORT – EHealth 13a, b | **13a: No EHealth-specific additions** |  | | |  |
|  |  |  | 13b | | For each group, losses and exclusions after randomisation, together with reasons | | | N/A |  | 13b (i) An attrition diagram (e.g., proportion of participants still logging in or using the intervention/comparator in each group plotted over time, similar to a survival curve) or other figures or tables demonstrating usage/dose/engagement | N/A | | |  |
| **ecruitment** | | | 14a | | Dates defining the periods of recruitment and follow-up | | | 11, 31 | CONSORT – EHealth 14a, b | 14a (i) Indicate if critical “secular events” [1] fell into the study period (e.g., significant changes in Internet resources available or “changes in computer hardware or Internet delivery resources) | N/A | | |  |
|  |  |  | 14b | | Why the trial ended or was stopped | | | N/A |  | **14b: No EHealth-specific additions** |  | | |  |
| **Baseline data** | | | 15 | | A table showing baseline demographic and clinical characteristics for each group | | | N/A | CONSORT – EHealth 15 | 15 (i) Report demographics associated with digital divide issues, such as age, education, gender, social-economic status, computer/Internet/ehealth literacy of the participants, if known. | N/A | | |  |
| **Numbers analysed** | | | 16 | | For each group, number of participants (denominator) included in each analysis and whether the analysis was by original assigned groups | | | N/A | CONSORT – EHealth 16 | 16 (i) Report multiple “denominators” and provide definitions. Always clearly define “use” of the intervention. | N/A | | |  |
|  |  |  |  |  |  |  |  |  |  | 16 (ii) Primary analysis should be intent-to-treat; secondary analyses could include comparing only “users”, with the appropriate caveats that this is no longer a randomized sample (see 18-i) | N/A | | |  |
| **Outcomes and estimation** | | | 17a | | For each primary and secondary outcome, results for each group, and the estimated effect size and its precision (such as 95% confidence interval) | | | N/A | CONSORT – EHealth 17a, b | 17a (i) In addition to primary/secondary (clinical) outcomes, the presentation of process outcomes such as metrics of use and intensity of use (dose, exposure) and their operational definitions is critical. This also to refers to more continuous exposure metrics such as “average session length”. | N/A | | |  |
|  |  |  | 17b | | For binary outcomes, presentation of both absolute and relative effect sizes is recommended | | | N/A |  | **17b: No EHealth-specific additions** |  | | |  |
| **Ancillary analyses** | | | 18 | | Results of any other analyses performed, including subgroup analyses and adjusted analyses, distinguishing pre-specified from exploratory | | | N/A | CONSORT – EHealth 18 | 18 (i) A subgroup analysis of comparing only users is not uncommon in ehealth trials, but if done it must be stressed that this is a self-selected sample and no longer an unbiased sample from a randomized trial | N/A | | |  |
| **Harms** | | | 19 | | All important harms or unintended effects in each group (for specific guidance see CONSORT for harms) | | | N/A | CONSORT – EHealth 19 | 19 (i) Include privacy breaches, technical problems. | N/A | | |  |
|  |  |  |  |  |  |  |  |  |  | 19 (ii) Include qualitative feedback from participants or observations from staff/researchers, if available, on strengths and shortcomings of the application, especially if they point to unintended/unexpected effects or uses. | N/A | | |  |
| **Discussion** | | | | | | | | | | | | | | |
| **Limitations** | | 20 | | | | Trial limitations, addressing sources of potential bias, imprecision, and, if relevant, multiplicity of analyses | | 16-17 | CONSORT – EHealth 20 | 20 (i) Typical limitations in ehealth trials: Participants in ehealth trials are rarely blinded. Ehealth trials often look at a multiplicity of outcomes, increasing risk for a Type I error. Discuss biases due to non-use of the intervention/usability issues, biases through informed consent procedures, unexpected events | 16-17 | | | |
| **Generalisability** | | 21 | | | | Generalisability (external validity, applicability) of the trial findings | | N/A | CONSORT – EHealth 21 | 21 (i) Generalizability to other populations: In particular, discuss generalizability to a general Internet population, outside of a RCT setting, and general patient population, including applicability of the study results for other organizations | N/A | | | |
|  |  |  |  |  |  |  |  |  |  | 21 (ii) Discuss elements in the RCT that would be different in a routine application setting (e.g., prompts/reminders, more human involvement, training sessions or other co-interventions) and what impact the omission of these elements could have on use, adoption, or outcomes if the intervention is applied outside of a RCT setting | N/A | | | |
| **Interpretation** | | 22 | | | | Interpretation consistent with results, balancing benefits and harms, and considering other relevant evidence | | N/A | CONSORT – EHealth 22 | 22 (i) Restate study questions and summarize the answers suggested by the data, starting with primary outcomes and process outcomes. | N/A | | | |
|  |  |  |  |  |  |  |  |  |  | 22 (ii) Highlight unanswered new questions, suggest future research | N/A | | | |
| **Other Information** | | | | | | | | | | | | | | |
| **Registration** | 23 | | | | | Registration number and name of trial registry | | 2, 15 | CONSORT – EHealth 23 | **23: No EHealth-specific additions** |  | | | |
| **Protocol** | 24 | | | | | Where the full trial protocol can be accessed, if available | | N/A | CONSORT – EHealth 24 | **24: No EHealth-specific additions** |  | | | |
| **Funding** | 25 | | | | | Sources of funding and other support (such as supply of drugs), role of funders | | 19 | CONSORT – EHealth 25 | **25: No EHealth-specific additions** |  | | | |

| Supplementary Table 3.  Acceptability scores by age and gender. | | | | | | | | | |
| --- | --- | --- | --- | --- | --- | --- | --- | --- | --- |
|  | *Girls* | | |  | *Boys* | | | *Whole Sample* | |
|  | *13 – 15 years old (M, SD)* | *16 – 18 years old*  *(M, SD)* |  | | | *13 – 15 years old (M, SD)* | *16 – 18 years old (M, SD)* | | *13 – 18 years old (M, SD)* |
| *Acceptability Items* |  |  |  | | |  |  | |  |
| Emotive Response | 6.65 (0.37) | 6. 32 (0.99) |  | | | 6.25 (0.98) | 5.63 (1.44) | | 6.25 (1.05) |
| 1. How interesting was your conversation with Dandara and/or Gabriel (e.g., the information, tasks and storyline)? | 6.66 (0.58) | 6.45 (0.92) |  | | | 6.29 (1.25) | 5.58 (1.85) | | 6.29 (1.25) |
| 2. How much did you enjoy talking with Dandara and/or Gabriel? | 6.86 (0.34) | 6.50 (0.98) |  | | | 6.15 (1.35) | 5.53 (1.83) | | 6.32 (1.27) |
| 3. How comfortable did you feel talking about your body image with Dandara and/or Gabriel? | 6.42 (0.73) | 6.02 (1.46) |  | | | 6.26 (1.02) | 5.84 (1.31) | | 6.15 (1.20) |
| Relevance | 6.52 (0.52) | 6.45 (0.77) |  | | | 6.35 (0.79) | 5.82 (1.35) | | 6.33 (0.92) |
| 4. Did the chatbot discuss things that were important to you? | 6.59 (0.83) | 6.68 (0.55) |  | | | 6.48 (0.8) | 6.06 (1.52) | | 6.49 (0.97) |
| 5. Did the chatbot discuss things that are important to other young people your age? | 6.72 (0.56) | 6.72 (0.82) |  | | | 6.66 (0.78) | 6.39 (1.10) | | 6.65 (0.82) |
| 6. How helpful was the chatbot at giving you skills to improve your body image? | 6.22 (0.98) | 5.95 (1.30) |  | | | 5.89 (1.42) | 5.10 (2.04) | | 5.86 (1.48) |
| Ease of Use | 6.24 (0.93) | 6.13 (0.91) |  | | | 6.23 (0.78) | 6.12 (0.91) | | 6.19 (0.88) |
| 7. How quickly did the chatbot address your questions or concerns? 8. How easy was the chatbot to use? 9. How accurate was the chatbot’s response to your questions or concerns? | 6.10 (1.2)  6.50 (0.94)  6.10 (1.2) | 6.11 (1.08)  6.30 (1.3)  5.98 (1.13) |  | | | 6.19 (1.24)  6.41 (1.37)  6.11 (1.15) | 6.23 (0.89)  6.40 (1.22)  5.73 (1.55) | | 6.15 (1.09)  6.41 (1.18)  6.0 (1.24) |
| Likelihood to Recommend/Re-engage | 6.63 (0.58) | 6.28 (1.13) |  | | | 6.04 (1.14) | 5.62 (1.63) | | 6.20 (1.19) |
| 10. Would you recommend this chatbot to a friend, who is worried about their body image? 11. Would you talk to this chatbot again, in the future? | 6.75 (0.60)  6.53 (0.91) | 6.50 (0.95)  6.08 (1.46) |  | | | 6.55 (1.01)  5.52 (1.74) | 6.06 (1.55)  5.17 (2.00) | | 6.49 (1.06)  5.92 (1.58) |
| Overall Acceptability (11 items) | 6.51 (0.45) | 6.30 (0.77) |  | | | 6.25 (0.75) | 5.74 (1.18) | | 6.23 (0.85) |

`


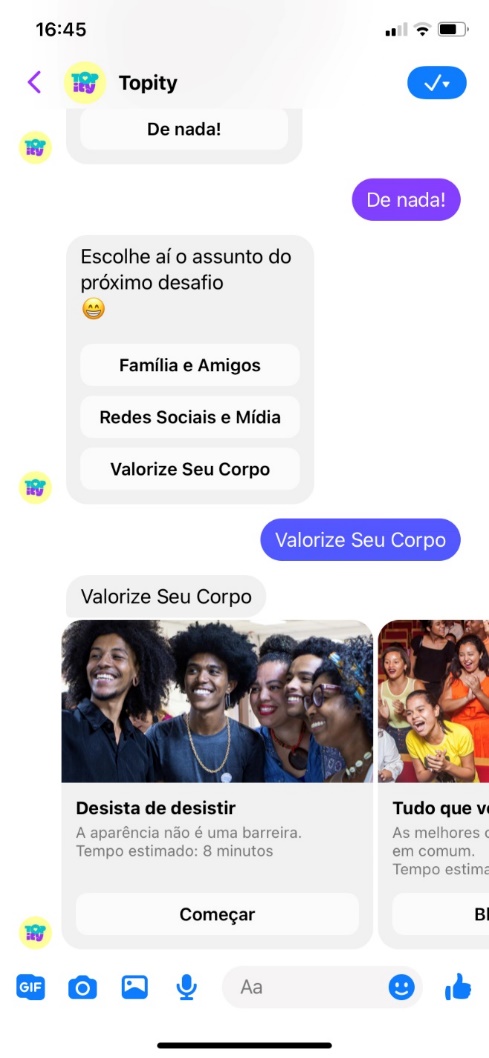

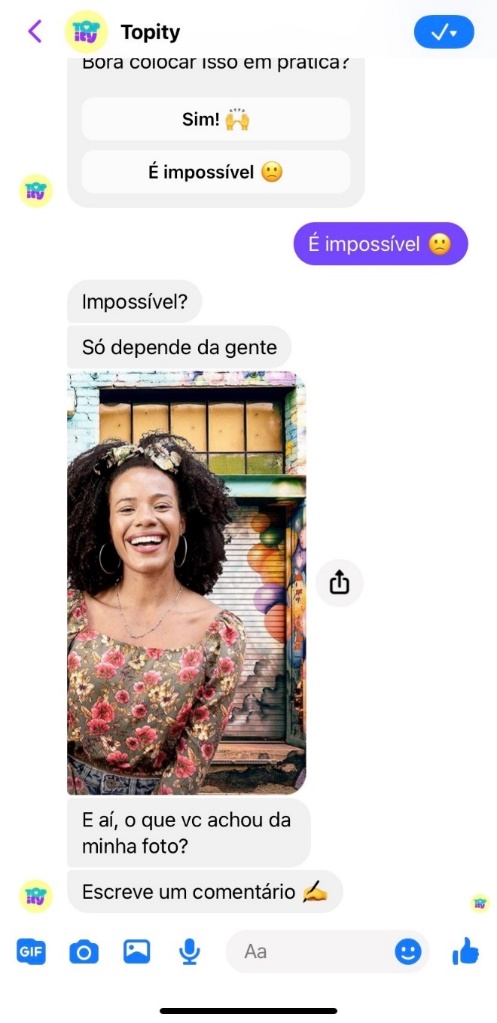

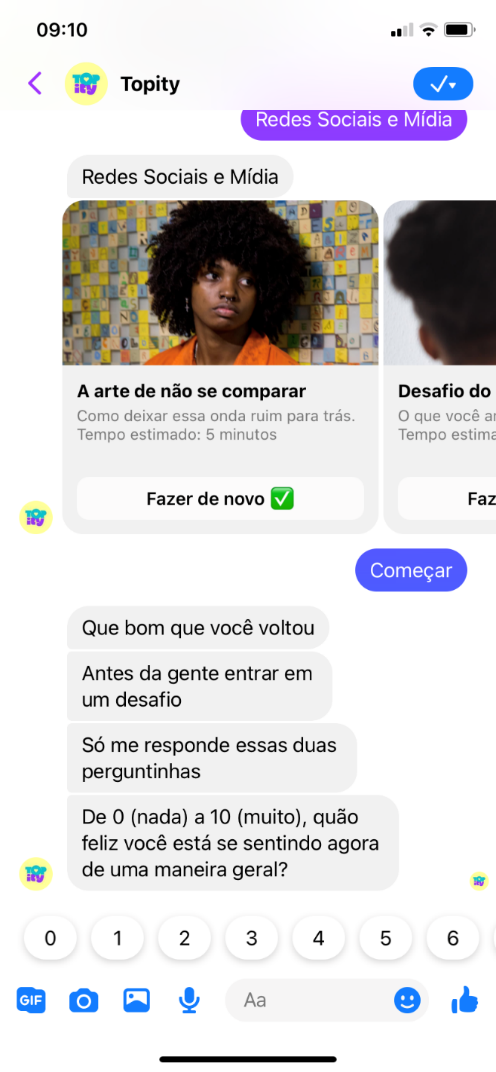


*Supplementary Fig 1.* Screenshots of a Topity conversation on mobile vie

Questionnaire Inventory

The current questionnaire inventory was developed by CAR to provide Toluna with a step-by-step guide for what needs to be included in the online surveys. The inventory comprises of two sections.

The first section is a flow diagram of the four surveys and their key elements. The second section is a full breakdown of each survey and the relevant information that is needed to be included at each timepoint (e.g., instructions, questions). Please note that **some questions are repeated at each time point** (e.g., body image, mood and self-efficacy outcome measures); however, there are also **unique** **pieces of information** that occur at each time point.

To aid the development of the online survey, CAR have put instructions to Toluna in **[red parentheses].** Where possible, CAR has provided instructions and questions in Brazilian Portuguese. We kindly ask that you do not change the wording of these as they have been purposefully translated in this way.

**Section One - Survey Outline**

**SURVEY ONE (Adolescents)**

**PARENTAL INFORMATION SHEET AND CONSENT**

**ADOLESCENT INFORMATION SHEET AND CONSENT**

**ADOLESCENT DEMOGRAPHICS**

(AGE, GENDER, ETHNICITY)

**PRE-INTERVENTION MEASURES**

(BODY IMAGE, MOOD, SELF-EFFICACY)

**RANDOMISATION TO GROUP**

**GROUP 2**

(Assessment only; control)

Assessment Only

**GROUP 1**

Chatbot

**POST-INTERVENTION MEASURES**

(BODY IMAGE, MOOD, SELF-EFFICACY)

**SURVEY FOUR (Adolescents)**

**SURVEY THREE (Adolescents)**

**SURVEY TWO (Adolescents)**

**1 WEEK FOLLOW UP MEASURES**

(BODY IMAGE, MOOD, SELF-EFFICACY)

**1 MONTH FOLLOW UP MEASURES**

(BODY IMAGE, MOOD, SELF-EFFICACY)

**Section Two - Survey Inventory.**

**PARENTAL INFORMATION SHEET AND CONSENT**

**[The survey content should be presented in an easy to read format for both parents and adolescents. Given the amount of information for parents to read, we suggest presenting this information across 2-3 pages]**

**[All headings should be in BOLD].**

**Information for Parents**

**Using a Chatbot to Help Brazilian Adolescents**

We invite your child to take part in an exciting research project that is being conducted by researchers at the Centre for Appearance Research (CAR), in collaboration with the Dove Self-Esteem Project (DSEP) and UNICEF. Before you consent to your child’s participation, it is important for you understand why this research is being conducted, what it will involve and the associated outcomes. Please read the following information carefully.

If you have any questions or would like more information please contact;

1. **Dr Emily Matheson**, Faculty of Health and Applied Science, University of the West of England, United Kingdom: [Emily.Matheson@uwe.ac.uk](mailto:Emily.Matheson@uwe.ac.uk) [English speaking only].
2. **Dr Ana Amaral**, Professora de Educação Física

IF Sudeste MG - Campus Barbacena, Brasil: [ana.amaral@ifsudestemg.edu.br](mailto:ana.amaral@ifsudestemg.edu.br) [Brazilian Portuguese speaking].

1. **Gabriela Goulart Mora** Oficial de Programa – Desenvolvimento e Participação de Adolescentes, UNICEF Brazil:[gmora@unicef.org](mailto:gmora@unicef.org) [Brazilian Portuguese speaking].

**Who is conducting the research?**

This research is being conducted by researchers at CAR, in collaboration with DSEP and UNICEF. The researchers at CAR specialize in young people’s mental health and well-being. They develop tools and programs that aim to help children and adolescents around the world to feel more confident about themselves and their appearance.

The CAR researchers include, Dr Emily Matheson, Ms Harriet Smith and Professor Phillippa Diedrichs. You can follow these link to access their professional information;

Dr Emily Matheson: <https://people.uwe.ac.uk/Person/EmilyMatheson>

Miss Harriet Smith: <https://people.uwe.ac.uk/Person/Harriet5Smith>

Professor Phillippa Diedrichs: <https://people.uwe.ac.uk/Person/PhillippaDiedrichs>

**What is the aim of this research?**

This project is looking at whether a new chatbot is effective at improving the body confidence and self-esteem of Brazilian adolescents aged 13-18 years old. A chatbot is an online computer program designed to mimic a real conversation. Our chatbot has been carefully designed by body image researchers, a technology company, adolescent advisors from UNICEF Brazil and members from DSEP. The chatbot will talk to adolescents about their body image concerns (e.g., how they feel and think about their body) and provide them with tools and strategies to help them feel more confident about themselves and their appearance.

**Why has my child been invited to take part?**

The chatbot aims to improve the body confidence and self-esteem amongst Brazilian adolescents aged 13-18 years. We identify your child as meeting these criteria, and therefore inviting them to participate in our research.

**What will this research involve?**

If you and your child agree to their participation in our research, your child will complete several activities over the course of **one month**. In the event that you consent to your child’s participation, they will receive an email from Toluna that will outline the current research and the tasks involved. Following their consent, your child will complete the following tasks:

- Phase 1: Survey 1 (15 minutes)
- Phase 2: Group one (Chatbot participation; unlimited use over 72 hours) or Group two (Assessment only; activities as usual)
- Phase 3: Survey 2 (15 minutes)
- Phase 4: Survey 3 (15 minutes)
- Phase 5: Survey 4 (15 minutes)

In each survey, your child will be asked to answer a series of questions that relate to mental health and well-being. Each survey should take no longer than 15 minutes to complete. Below is a sample of the questions:

Example question 1: *‘How happy do you feel, right now?’*

Example question 2: *‘Would you recommend this chatbot to a friend?’*

Example question 3: *‘How confident do you feel that you can carry out daily activities to improve your body image?’*

After ‘**Survey 1**’, adolescents will be **randomly** assigned into one of two groups. One group will be invited to engage with the chatbot over a 72-hour period, while the second group will go about their normal activities and **not engage** with the chatbot. At the end of the research, adolescents who did not engage with the chatbot will be invited to do so.

Adolescents who were invited to engage with the chatbot, will be encouraged to independently interact with the chatbot as much as they need and/or want over a 72-hour period.

After the 72-hour period adolescents in both groups will be asked to complete ‘**Survey 2**’. One week after this survey, all adolescents will complete ‘**Survey 3**’ and then one-month after this survey, all adolescents will complete ‘**Survey 4**’.

Your child participation in this project will be entirely online. Your child will receive a small monetary reimbursement for their participation, which will cover the cost of their internet usage used during this research project. After the completion of all stages, Toluna will send your child an e-voucher via e-mail.

**Do I need to help my child complete the study?**

The questions in the surveys have been carefully designed and tested with Brazilian adolescents. We encourage that your child completes the surveys privately and independently. However, if your child requires any assistance with comprehension (e.g. they do not understand a word or instruction) then we encourage you to provide passive support (e.g., define the word or explain the instructions). Please do not answer the questions for your child or guide them to answer in a particular way.

**What are the benefits of taking part in this study?**

Firstly, to date, our research into young people’s mental health indicates that interacting with online content that relates to body confidence and self-esteem is a positive experience. By participating, your child may experience direct benefits. For example, learning about important issues for young people in Brazil. Second, the results of this research will also help to inform future research into mental health tools for adolescents in Brazil and across the world. Lastly, your child will be provided with a small monetary compensation for their participation.

**Are there any disadvantages of taking part?**

We do not foresee that participation in this research will cause harm to your child. However, in the unlikely event that this occurs, please notify either Toluna or our research team (details provided above), who will provide yourself and/or your child with a list of resources that provide support to children in Brazil. This list of resources will also appear at the end of each survey.

At the beginning of the study (**Survey 1**), adolescents will be presented with a similar information sheet, which will reassure your child that their answers are confidential and that there are no right or wrong answers. They will be encouraged to answer honestly. Your child will also be advised that they can withdraw from the study at any point, and that they will receive the credit for completed activities up to the point of withdrawing from the research.

**What data do we collect?**The data we collect will vary from project to project. Researchers will only collect data that is essential for their project.

Personal data collected in this project will include; your child’s gender, your child’s age, your child’s ethnicity and their region of residence. Your child may be required to create a unique ID code, which will allow our researchers to match adolescents’ data across the four surveys.

**What will happen to your child’s information?**

Information provided by your child will be kept confidential and pseudo-anonymized (i.e., your child will be identified by their unique ID, not their full name). All data will be kept in on a secure university hard drive that only the researchers can access; this is in line with the United Kingdom Data Protection Act 2018.

We will keep your child’s data for as long as is necessary to fulfil the cited purpose of the research. The length of time we keep personal data will depend on several factors including the significance of the data, funder requirements, and the nature of the study. Anonymized data that falls outside the scope of data protection legislation as it contains no identifying or identifiable information may be stored in the university’s research data archive or another carefully selected appropriate data archive.

For more information on the how your data is protected, please refer to the **Privacy Notice**, which accompanies this information sheet.

**Who do we share your data with?**

Personal data collected during this study **will not** be shared with anyone. Only the primary researcher and co-researchers will have access to the data that is collected. All data will be anonymized before analysis.

**Where will the results of the research study be published?**

Key findings from this research study may be shared both within and outside the University of the West of England. Data will be cited in stakeholder reports and/or academic peer reviewed journal articles and at national (UK and Brazil) and international conferences. Lastly, we will develop parent and child-friendly materials for the wider Brazilian community, which provides a summary of the findings.

**Who has ethically approved this research?**

This research project has been reviewed and approved by the Faculty of Health and Applied Science, at the University of the West of England Research Ethics Committee and the Ethic Committee at Federal Institute of Education, Science and Technology of Southeast of Minas Gerais and the Brazilian National Committee for Ethics in Research (CONEP).

Any comments, questions or complaints about the ethical conduct of this study can be addressed to:

- **Research Ethics Committee at the University of the West of England** at [Researchethics@uwe.ac.uk](mailto:Researchethics@uwe.ac.uk) [Idioma: Ingles/English]
- **Comitê de Ética em Pesquisa com Seres Humanos do Instituto Federal de Educação, Ciência e Tecnologia do Sudeste de Minas Gerais** através do e-mail: [etica.pesquisa@ifsudestemg.edu.br](mailto:etica.pesquisa@ifsudestemg.edu.br) [Idioma: Português/Portuguese].
- **Comissão Nacional de Ética em Pesquisa – (CONEP):** SRTVN - Via W 5 Norte - Edifício PO700 - Quadra 701, Lote D - 3º andar - Asa Norte, CEP 70750 -521, Brasília (DF); Telefone: (61) 3315-5877. Horário de atendimento: 08h às 18h.

**What if something goes wrong?**

Should you or your child raise any concerns or complaints as a result of the research, we ask that you contact the principle researcher (Dr Emily Matheson) or one of our Brazilian collaborator(s):

1. **Dr Emily Matheson**, Faculty of Health and Applied Science, University of the West of England, United Kingdom: [Emily.Matheson@uwe.ac.uk](mailto:Emily.Matheson@uwe.ac.uk) [English speaking only].
2. **Dr Ana Amaral**, Professora de Educação Física

IF Sudeste MG - Campus Barbacena, Brasil: [ana.amaral@ifsudestemg.edu.br](mailto:ana.amaral@ifsudestemg.edu.br) [Brazilian Portuguese speaking].

1. **Gabriela Goulart Mora** Oficial de Programa – Desenvolvimento e Participação de Adolescentes, UNICEF Brazil:[gmora@unicef.org](mailto:gmora@unicef.org) [Brazilian Portuguese speaking].

**What if I have more questions or do not understand something?**

If you would like further information about the research, please contact the principle researcher (Dr Emily Matheson) or one of our Brazilian collaborator(s):

1. **Dr Emily Matheson**, Faculty of Health and Applied Science, University of the West of England, United Kingdom: [Emily.Matheson@uwe.ac.uk](mailto:Emily.Matheson@uwe.ac.uk) [English speaking only].
2. **Dr Ana Amaral**, Professora de Educação Física

IF Sudeste MG - Campus Barbacena, Brasil: [ana.amaral@ifsudestemg.edu.br](mailto:ana.amaral@ifsudestemg.edu.br) [Brazilian Portuguese speaking].

1. **Gabriela Goulart Mora** Oficial de Programa – Desenvolvimento e Participação de Adolescentes, UNICEF Brazil:[gmora@unicef.org](mailto:gmora@unicef.org) [Brazilian Portuguese speaking].

**Consent**

Please ensure that you have read and understood the above information. If you are happy for your child to take part in this research study, please indicate by selecting the appropriate box:

[I consent]

[I **do not** consent]

**[Page Break]**

**[‘I consent’ page]**

Thank you for **consenting** to your child’s participation in this research.

**[If the parent selects ‘I consent’ they will also need to provide a digital signature, which is then verified by DocUSign]**

Thank you. We will be in contact with your child via e-mail within the next 24 hours, to provide them with a link to Survey 1.

**[‘I do not consent’ page]**

Thank you for taking the time to learn about our research. You have **not given consent** for your child to participate in the research and therefore we will **not contact** your child.

If you feel comfortable, please can you advise why you do not wish for your child to participate?

**ADOLESCENT INFORMATION SHEET AND CONSENT**

**[Please include this graphic at the beginning of the survey, this signifies the start of the survey for adolescents].**


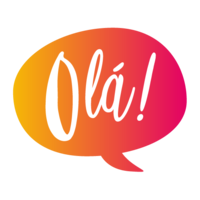


**Information Sheet for Participants**

**Using a Chatbot to Help Brazilian Adolescents**

Your parent has indicated that you might like to participate in our research study! The information below will tell you all about the research and what you’ll be asked to do.

Once you have read the below information, you can decide if you would like to take participate.

**Who is conducting this research?**

The project is being conducted by researchers at the Centre for Appearance Research at the University of the West of England, United Kingdom.

If you have any questions or would like more information about the research, please contact either one of these people below:

1. **Dr Emily Matheson**, Faculty of Health and Applied Science, University of the West of England, United Kingdom: [Emily.Matheson@uwe.ac.uk](mailto:Emily.Matheson@uwe.ac.uk) [English speaking only].
2. **Dr Ana Amaral**, Professora de Educação Física

IF Sudeste MG - Campus Barbacena, Brasil: [ana.amaral@ifsudestemg.edu.br](mailto:ana.amaral@ifsudestemg.edu.br) [Brazilian Portuguese speaking].

1. **Gabriela Goulart Mora** Oficial de Programa – Desenvolvimento e Participação de Adolescentes, UNICEF Brazil:[gmora@unicef.org](mailto:gmora@unicef.org) [Brazilian Portuguese speaking].

**What is this project about?**

This project is looking at ways to support young people in Brazil to be happy and healthy. We are trying to understand whether a chat-bot can help adolescents to feel more confident about themselves.

A chatbot is a computer program that is similar to a conversation with a friend. Our chatbot is not a real person, but it has been carefully designed to talk with Brazilian adolescents about body confidence and self-esteem!

**What will I be doing?**

If you wish to participate in our research, you will be asked to engage in several activities over the next month. Every young person that participates will be asked to complete **4 surveys over the next month**. These surveys will ask about your mental health and well-being, and should take no longer than 15 minutes of your time. A research agency named Toluna, will send you a link to each of the survey via email.

At the end of the first survey, some adolescents will be invited to engage with the chatbot, meanwhile other adolescents will be asked to go about their normal activities and will not engage with the chatbot. **Don’t worry**, young people who do not interact with the chatbot during our research, will be invited to do so, after the research is complete.

The chatbot will be hosted on Facebook Messenger. The young people who are invited to interact with the chatbot, will interact with the bot for as long as they need or want over 72-hours.

When you participate in the research, there are no right or wrong answers. We are very interested in your opinion; therefore, please be honest when answer survey questions and interacting with the chatbot!

Every young person who participates in the research will receive a small monetary compensation to cover the cost of your internet usage. You will receive a small fee for each survey that you complete and then a final fee when you complete all activities.

**When will this happen?**

Once you have read through this information, you may continue to the next page where you will receive further instructions on how to begin the study.

**Who will see my results?**

All of your answers will be kept private. Your data will not contain identifying information (e.., your name or location). You will be provided with a unique ID. This ID will help our researchers to match your responses across the four surveys. Only the researchers will see your data.

When the research is finished, the results will be used to understand if the chat-bot helped young people, like yourself. We may publish our findings in a written report. This report may be read by people in Brazil and across the world. If we do this, the information will not contain any personal information of our participants.

**Do I have to take part in this study?**

Your parent/guardian has confirmed that they are happy for you to participate. However, you **do not** have to participate if you do not want to. If you agree to participate, but you change your mind, you can withdraw from the study at any point. You also do not need to tell anyone why you dropped out.

**Thank you vouchers.**

Every young person who participates in the research will receive a small monetary compensation for their time. You will receive a small fee for each survey that you complete and then a final fee when you complete all activities.

Toluna will contact you via e-mail with the details of how to receive this voucher at the very end of the project!

**[INSERT ‘NEXT’ OR ‘>>’ BUTTON]**

**[PAGE BREAK]**

**ADOLESCENT CONSENT TO PARTICIPATE**

**[Please use a forced response option for this question]**

Thank you for reading the information about this project! Please indicate whether or not you would like to participate:

**YES – I would like to take part.**

**NO – I do not want to take part.**

**[If the “YES” box is selected – present the following message]**

Great! You are ready to begin the survey. Please press the ‘next’ button to start.

**[If “NO” is selected – provide a prompt message]**

Are you sure that you **do not** want to participate in this project?

**[Present two response options:]**

- Yes, that’s right, I **do not want** to participate
- No, my mistake, I **would like** to participate

**[If “NO” is selected – present the following message]**

Great! You are ready to begin the survey. Please press the ‘next’ button to start.

**[If “YES” is selected – present the following message]**

Thank you for reading about our research. We understand that on this occasion, you do not wish to participate in the research.

If you feel comfortable, please can you advise why you do not wish to participate?

If you need further support for your mental health and well-being, as a first step we would encourage you to reach out to a trusted family member or friend discuss your concerns.

You can also access mental health and well-being support services, using the below links:

**Apoio Nacional**

Os seguintes links são boas fontes de apoio para qualquer pessoa procurando ajuda e conselhos confidenciais:

- **Transtornos Alimentares:**
  - ASTRAL – Associação Brasileira de Transtornos Alimentares: <https://astralbr.org/buscar-ajuda/>

**Apoio Local**

- **Transtornos Alimentares:**
  - AMBULIM: [http://www.ambulim.org.br](http://www.ambulim.org.br/) [São Paulo]
  - PROATA: [https://www.proata.com.br/](https://eur01.safelinks.protection.outlook.com/?url=https%3A%2F%2Fwww.proata.com.br%2F&data=02%7C01%7CHarriet5.Smith%40uwe.ac.uk%7Cc2d2059b611749600e1f08d81c32d810%7C07ef1208413c4b5e9cdd64ef305754f0%7C0%7C0%7C637290351984265808&sdata=RPgwjnOIhQ27Zq88cjG5XTzQGqB9MiWdWOmVjZrG4dI%3D&reserved=0) [São Paulo]

Thank you again for your interest in this project, you may exit the survey.

**[INSERT ‘NEXT’ OR ‘>>’ BUTTON]**

**[PAGE BREAK]**

**[Include this graphic]**

**DEMOGRAPHICS QUESTIONS – 1 MIN**


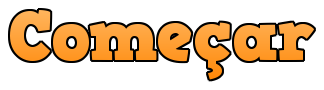


**This survey is going to ask you questions about your everyday experiences, thoughts and feelings.**

**First, we are going to ask some questions about you…**

1. **What gender are you? [one response selection only]**
   - Boy
   - Girl
   - Other (please specify) [provide text-box]
2. **How old are you? [one response selection only]**
   - 13
   - 14
   - 15
   - 16
   - 17
   - 18
   - 19+
3. **How would you describe yourself? [ethnicity questions]**
   - Asiatica (Asian)
   - Branca (White)
   - Indigena (Indigenous)
   - Parda (Black)
   - Preto (Mixed ethnicity)
   - Outra -please specify - **[free entry text box here]**

**[INSERT ‘NEXT’ OR ‘>>’ BUTTON]**

**[PAGE BREAK]**

1. **In which region of Brazil, do you live? [one response selection only]**
   - North
   - Northeast
   - Central West
   - Southeast
   - South
   - I don’t know
2. **In which State/City of Brazil, do you live? [one response selection only]**
   - Acre
   - Alagoas
   - Amazonas
   - Amapá
   - Bahia
   - Ceará
   - Distrito Federal
   - Espírito Santo
   - Goiás
   - Maranhão
   - Mato Grosso
   - Mato Grosso do Sul
   - Minas Gerais
   - Pará
   - Paraíba
   - Paraná
   - Piauí
   - Pernambuco
   - Rio Grande do Norte
   - Rio Grande do Sul
   - Rio de Janerio
   - Rondônia
   - Roraima
   - São Paulo
   - Santa Catarina
   - Sergipe
   - Tocantins
   - I don’t know

**Great! Please continue to the next page.**

**[INSERT ‘NEXT’ OR ‘>>’ BUTTON]**

**[PAGE BREAK]**

**PRE-INTERVENTION MEASURES**

(BODY IMAGE, MOOD, SELF-EFFICACY)

**Please read the instructions carefully and answer each question below. Remember, there are no right or wrong answers. We are interested in your opinions!**

**Let’s start with an example question…**

**Example Question**

**This question is rated from 1 to 10. Circling 1, means your really don’t enjoy spending time with your family. Circling 5, means you don’t mind spending time with them. Circling 10, means you really enjoy spending time with your family.**

**Circle a number from 1 to 10 that represents how you feel about spending time with your family**

**Using the scale, please indicate how you feel about the below statement.**

I enjoy spending time with my family.

1 2 3 4 5 6 7 8 9 10

Not At All Very Much

**[INSERT ‘NEXT’ OR ‘>>’ BUTTON]**

**[PAGE BREAK]**

**Excellent! Now that you are familiar with the question and the answer format, we would like to ask you some more questions about your everyday experiences.**

**Remember, there are no right or wrong** **answers.**

**[INSERT ‘NEXT’ OR ‘>>’ BUTTON]**

**[PAGE BREAK]**

**[BODY ESTEEM MEASURE – to be presented in BR P]**

**Using the scale below, please mark how often each of the following statements applies to you.**

|  | Never  1 | Rarely  2 | Sometimes  3 | Often  4 | Always  5 |
| --- | --- | --- | --- | --- | --- |
| 1. I like how I look in photos. |  |  |  |  |  |
| 1. Other people consider me beautiful. |  |  |  |  |  |
| 1. I am proud of my body. |  |  |  |  |  |
| 1. I am preoccupied with trying to change my weight. |  |  |  |  |  |
| 1. I think my looks would help me to get a job. |  |  |  |  |  |
| [INSERT ‘NEXT’ OR ‘>>’ BUTTON]  [PAGE BREAK] | | | | |  |
| 1. I like what I see in the mirror. |  |  |  |  |  |
| 1. I would change lots of things about my appearance if I could. |  |  |  |  |  |
| 1. I am satisfied (happy) with my weight. |  |  |  |  |  |
| 1. I would like to be more beautiful. |  |  |  |  |  |
| 1. I really like how much I weigh. |  |  |  |  |  |
| 1. I would like to look like someone else. |  |  |  |  |  |
| [INSERT ‘NEXT’ OR ‘>>’ BUTTON]  [PAGE BREAK] | | | | |  |
| 1. People my own age like my appearance/the way I look. |  |  |  |  |  |
| 1. For this item, please select ‘Às vezes’ |  |  |  |  |  |
| 1. My appearance makes me sad. |  |  |  |  |  |
| 1. I am as beautiful as most people. |  |  |  |  |  |
| 1. I am really happy with how I look/my appearance. |  |  |  |  |  |
| 1. I have the right weight for my height. |  |  |  |  |  |
| 1. I feel ashamed of my appearance. |  |  |  |  |  |
| [INSERT ‘NEXT’ OR ‘>>’ BUTTON]  [PAGE BREAK] | | | | | |
| 1. When I weight myself I get depressed (sad). |  |  |  |  |  |
| 1. My weight makes me unhappy. |  |  |  |  |  |
| 1. My appearance helps me get romantic dates. |  |  |  |  |  |
| 1. I get distressed about my appearance. |  |  |  |  |  |
| 1. I think I have a good body. |  |  |  |  |  |
| 1. I am as beautiful as I would like to be. |  |  |  |  |  |

**[INSERT ‘NEXT’ OR ‘>>’ BUTTON]**

**[PAGE BREAK]**

**[FILLER ITEMS - to be presented in BR P]**

**Using the scale, please indicate how you feel about each statement…**

- **Spending time with my friends is important to me.**

1 2 3 4 5 6 7 8 9 10
Not at all Very much

- **I enjoy being physically active.**

1 2 3 4 5 6 7 8 9 10
Not at all Very much

- **Having a good sleep routine is important to me.**

1 2 3 4 5 6 7 8 9 10
Not at all Very much

**[INSERT ‘NEXT’ OR ‘>>’ BUTTON]**

**[PAGE BREAK]**

**[MOOD MEASURE – to be presented in BR P]**

**Please indicate how much you felt this way in the past week…**

|  | Not even a little  1 | A little  2 | So-so  3 | Quite  4 | Very much  5 |
| --- | --- | --- | --- | --- | --- |
| Divertido (fun) |  |  |  |  |  |
| Alegre (joyful) |  |  |  |  |  |
| Contente (content) |  |  |  |  |  |
| Animado (cheerful) |  |  |  |  |  |
| Incomodado (bothered) |  |  |  |  |  |
| Humilhado (humiliated) |  |  |  |  |  |
| Magoado (hurt) |  |  |  |  |  |
| Irritado (irritated) |  |  |  |  |  |

**[INSERT ‘NEXT’ OR ‘>>’ BUTTON]**

**[PAGE BREAK]**

**[FILLER ITEMS - to be presented in BR P]**

**Using the scale, please indicate how you feel about each statement…**

- **Doing well at school is important to me.**

1 2 3 4 5 6 7 8 9 10
Not at all Very much

- **Please select the number 8.**

1 2 3 4 5 6 7 8 9 10

Not at all Very much

- **I enjoy trying new things.**

1 2 3 4 5 6 7 8 9 10
Not at all Very much

- **Getting along with other people is important to me.**

1 2 3 4 5 6 7 8 9 10
Not at all Very much

**[INSERT ‘NEXT’ OR ‘>>’ BUTTON]**

**[PAGE BREAK]**

**[BODY IMAGE SELF-EFFICACY MEASURE - to be presented in BR P]**

*The term ‘body image’ takes into account your thoughts, feelings and behaviours in relation to your body. That is, it does not only refer to its appearance. Takes this into account when answering the scale below.*

Please rate your level of confidence for each item on the scale from 0-100

| 1. **I am able to learn and practice new skills to help improve my body image**   0 100  Not confident at all Moderately confident Very confident |
| --- |
| 1. **I am able to recognize situations that will worsen my body image***.*   0 100  Not confident at all Moderately confident Very confident |
| 1. **I am able to perform daily activities that improve my body image.**  0 100  Not confident at all Moderately confident Very confident |

**[INSERT ‘NEXT’ OR ‘>>’ BUTTON]**

**[PAGE BREAK]**

| 1. **I am able to confront people who worsen my body image.**  0 100  Not confident at all Moderately confident Very confident |
| --- |
| 1. **I am able to ask other people for help when I cannot improve my body image.**   0 100   Not confident at all Moderately confident Very confident |

**[INSERT ‘NEXT’ OR ‘>>’ BUTTON]**

**[PAGE BREAK]**

**RANDOMISATION**

**[At this stage, adolescents have completed all baseline measures. Now they need to be randomized into either the Intervention (Group 1) or the Control (Group 2) groups]**

**[Present the following message to GROUP 1 only]**


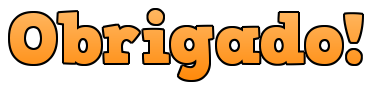


Thank you for answering our questions! In 24 hours, you will receive a link via email that will allow you to access the chatbot via Facebook Messenger.

We recommend that you **use the chatbot** **as often as you need or want** over the 72-hour period. The **more you engage** with the chatbot, the **better** your **confidence** and **self-esteem** is likely to be.

Many young people in Brazil report feeling sad or unhappy about how they look. Our chatbot uses special technology to speak with young people about their body image concerns. You will be able choose between two chatbot characters, either Dandara or Gabriel. Dandara and Gabriel are not real people; they are robots that have been carefully designed to help young people discuss their worries. When you talk to Dandara or Gabriel, they will provide you with tools and strategies that may help you, or someone you know, about concerns related to appearance.

Once you receive the link to the chatbot, you can use it as much as you wish. Remember, the **more you engage** with the chatbot, the **better** your **confidence** and **self-esteem** is likely to be. Three days after using the chatbot, we will send you a second survey, which will have similar questions to those you answered today. Please pay close attention to the topics and tasks discussed in your conversation with the chatbot, as we would like your feedback on your experience.

If you **do not** receive an e-mail with the link to the chatbot in the **next 24 hours**, please contact **[insert Toluna contact].**

**Fontes de apoio para os jovens.**

Se você foi afetado por qualquer assunto levantado durante o preenchimento desse questionário, sugerimos inicialmente que você procure um familiar ou amigo de confiança para obter apoio ou discutir qualquer dúvida ou preocupação em relação ao seu bem-estar.

Se você sentir que isso não é possível ou se sentir mais confortável em procurar apoio em outro lugar, veja abaixo uma lista de serviços nacionais e locais que podem ser capazes de ajudar.

**Apoio Nacional**

Os seguintes links são boas fontes de apoio para qualquer pessoa procurando ajuda e conselhos confidenciais:

- **Transtornos Alimentares:**
  - ASTRAL – Associação Brasileira de Transtornos Alimentares: <https://astralbr.org/buscar-ajuda/>

**Apoio Local**

- **Transtornos Alimentares:**
  - AMBULIM: [http://www.ambulim.org.br](http://www.ambulim.org.br/) [São Paulo]
  - PROATA: [https://www.proata.com.br/](https://eur01.safelinks.protection.outlook.com/?url=https%3A%2F%2Fwww.proata.com.br%2F&data=02%7C01%7CHarriet5.Smith%40uwe.ac.uk%7Cc2d2059b611749600e1f08d81c32d810%7C07ef1208413c4b5e9cdd64ef305754f0%7C0%7C0%7C637290351984265808&sdata=RPgwjnOIhQ27Zq88cjG5XTzQGqB9MiWdWOmVjZrG4dI%3D&reserved=0) [São Paulo]

**[Present the following message to GROUP 2 only]**


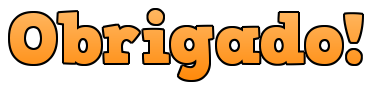


Thank you for answering our questions, we really appreciate your help!

At this stage, you won’t be talking with the chatbot. However, in the next few days, you will be sent a link to complete another survey. This survey will be similar to the one you completed today and should only take 15 minutes to complete. You should complete the survey independently, in private – just like you did today. Remember, there are no right or wrong answers to the questions you will be asked so please be as honest as possible!

Once you have completed all four survey, we will provide you with instructions on how you can access the chatbot, should you wish to do so!

In the meantime, please go about your everyday activities and look out for an email!

**Fontes de apoio para os jovens.**

Se você foi afetado por qualquer assunto levantado durante o preenchimento desse questionário, sugerimos inicialmente que você procure um familiar ou amigo de confiança para obter apoio ou discutir qualquer dúvida ou preocupação em relação ao seu bem-estar.

Se você sentir que isso não é possível ou se sentir mais confortável em procurar apoio em outro lugar, veja abaixo uma lista de serviços nacionais e locais que podem ser capazes de ajudar.

**Apoio Nacional**

Os seguintes links são boas fontes de apoio para qualquer pessoa procurando ajuda e conselhos confidenciais:

- **Transtornos Alimentares:**
  - ASTRAL – Associação Brasileira de Transtornos Alimentares: <https://astralbr.org/buscar-ajuda/>

**Apoio Local**

- **Transtornos Alimentares:**
  - AMBULIM: [http://www.ambulim.org.br](http://www.ambulim.org.br/) [São Paulo]
  - PROATA: [https://www.proata.com.br/](https://eur01.safelinks.protection.outlook.com/?url=https%3A%2F%2Fwww.proata.com.br%2F&data=02%7C01%7CHarriet5.Smith%40uwe.ac.uk%7Cc2d2059b611749600e1f08d81c32d810%7C07ef1208413c4b5e9cdd64ef305754f0%7C0%7C0%7C637290351984265808&sdata=RPgwjnOIhQ27Zq88cjG5XTzQGqB9MiWdWOmVjZrG4dI%3D&reserved=0) [São Paulo]

**[End of Survey 1]**

**[Start of Survey 2]**

**POST-INTERVENTION MEASURES**

(BODY IMAGE, MOOD, SELF-EFFICACY)

**SURVEY TWO**

**[Present the following message to adolescent in GROUP 1 only]:**


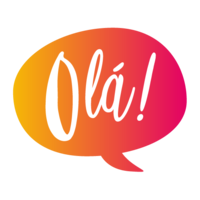


Welcome back! We hope you enjoyed interacting with the chatbot. We would like to ask you some questions about your experience of talking to either Dandara and/or Gabriel.

**Firstly, were you able to access the chatbot via Facebook Messenger?**

- Sim (yes)
- Não (no)

**[If ‘sim’ is selected – present the following message]**

That’s awesome! We hope it was a positive experience.

**Please continue to the next page to START the survey!**

**[If ‘não’ is selected – present the following message]**

Oh no! Please can you explain why you weren’t able to access the chatbot

**[INSERT ‘NEXT’ OR ‘>>’ BUTTON]**

**[PAGE BREAK]**

**[Present the following message to adolescents in GROUP 2 ONLY]:**


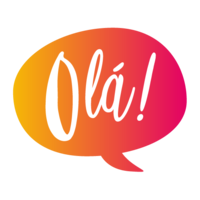


Welcome back! Thank you for all of your help so far. Today you will be completing your second survey, which should take approximately 15 minutes.

Remember that there are no right or wrong answers. Please complete this survey on your own, in private.

Continue to the next page, when you are ready to get started!

**[INSERT ‘NEXT’ OR ‘>>’ BUTTON]**

**[PAGE BREAK]**

**[Participants in both groups should be directed to this page [BODY ESTEEM MEASURE], following the introduction page]**

**[BODY ESTEEM MEASURE - to be presented in BR P]**

**Using the scale below, please mark how often each of the following statements applies to you.**

|  | Never  1 | Rarely  2 | Sometimes  3 | Often  4 | Always  5 |
| --- | --- | --- | --- | --- | --- |
| 1. I like how I look in photos. |  |  |  |  |  |
| 1. Other people consider me beautiful. |  |  |  |  |  |
| 1. I am proud of my body. |  |  |  |  |  |
| 1. I am preoccupied with trying to change my weight. |  |  |  |  |  |
| 1. I think my looks would help me to get a job. |  |  |  |  |  |
| [INSERT ‘NEXT’ OR ‘>>’ BUTTON]  [PAGE BREAK] | | | | |  |
| 1. I like what I see in the mirror. |  |  |  |  |  |
| 1. I would change lots of things about my appearance if I could. |  |  |  |  |  |
| 1. I am satisfied (happy) with my weight. |  |  |  |  |  |
| 1. I would like to be more beautiful. |  |  |  |  |  |
| 1. I really like how much I weigh. |  |  |  |  |  |
| 1. I would like to look like someone else. |  |  |  |  |  |
| [INSERT ‘NEXT’ OR ‘>>’ BUTTON]  [PAGE BREAK] | | | | |  |
| 1. People my own age like my appearance/the way I look. |  |  |  |  |  |
| 1. For this item, please select ‘Às vezes’ |  |  |  |  |  |
| 1. My appearance makes me sad. |  |  |  |  |  |
| 1. I am as beautiful as most people. |  |  |  |  |  |
| 1. I am really happy with how I look/my appearance. |  |  |  |  |  |
| 1. I have the right weight for my height. |  |  |  |  |  |
| 1. I feel ashamed of my appearance. |  |  |  |  |  |
| [INSERT ‘NEXT’ OR ‘>>’ BUTTON]  [PAGE BREAK] | | | | | |
| 1. When I weight myself I get depressed (sad). |  |  |  |  |  |
| 1. My weight makes me unhappy. |  |  |  |  |  |
| 1. My appearance helps me get romantic dates. |  |  |  |  |  |
| 1. I get distressed about my appearance. |  |  |  |  |  |
| 1. I think I have a good body. |  |  |  |  |  |
| 1. I am as beautiful as I would like to be. |  |  |  |  |  |

**[INSERT ‘NEXT’ OR ‘>>’ BUTTON]**

**[PAGE BREAK]**

**[FILLER ITEMS - to be presented in BR P]**

**Using the scale, please indicate how you feel about each statement…**

- **Spending time with my friends is important to me.**

1 2 3 4 5 6 7 8 9 10
Not at all Very much

- **I enjoy being physically active.**

1 2 3 4 5 6 7 8 9 10
Not at all Very much

- **Having a good sleep routine is important to me.**

1 2 3 4 5 6 7 8 9 10
Not at all Very much

**[INSERT ‘NEXT’ OR ‘>>’ BUTTON]**

**[PAGE BREAK]**

**[MOOD MEASURE - to be presented in BR P]**

**Please indicate how much you felt this way in the past week…**

|  | Not even a little  1 | A little  2 | So-so  3 | Quite  4 | Very much  5 |
| --- | --- | --- | --- | --- | --- |
| Divertido (fun) |  |  |  |  |  |
| Alegre (joyful) |  |  |  |  |  |
| Contente (content) |  |  |  |  |  |
| Animado (cheerful) |  |  |  |  |  |
| Incomodado (bothered) |  |  |  |  |  |
| Humilhado (humiliated) |  |  |  |  |  |
| Magoado (hurt) |  |  |  |  |  |
| Irritado (irritated) |  |  |  |  |  |

**[INSERT ‘NEXT’ OR ‘>>’ BUTTON]**

**[PAGE BREAK]**

**[FILLER ITEMS - to be presented in BR P]**

**Using the scale, please indicate how you feel about each statement…**

- **Doing well at school is important to me.**

1 2 3 4 5 6 7 8 9 10
Not at all Very much

- **Please select the number 8.**

1 2 3 4 5 6 7 8 9 10

Not at all Very much

- **I enjoy trying new things.**

1 2 3 4 5 6 7 8 9 10
Not at all Very much

- **Getting along with other people is important to me.**

1 2 3 4 5 6 7 8 9 10
Not at all Very much

**[INSERT ‘NEXT’ OR ‘>>’ BUTTON]**

**[PAGE BREAK]**

**[BODY IMAGE SELF-EFFICACY MEASURE - to be presented in BR P]**

*The term ‘body image’ takes into account your thoughts, feelings and behaviours in relation to your body. That is, it does not only refer to its appearance. Takes this into account when answering the scale below.*

Please rate your level of confidence for each item on the scale from 0-100

| 1. **I am able to learn and practice new skills to help improve my body image**   0 100  Not confident at all Moderately confident Very confident |
| --- |
| 1. **I am able to recognize situations that will worsen my body image***.*   0 100  Not confident at all Moderately confident Very confident |
| 1. **I am able to perform daily activities that improve my body image.**  0 100  Not confident at all Moderately confident Very confident |

**[INSERT ‘NEXT’ OR ‘>>’ BUTTON]**

**[PAGE BREAK]**

| 1. **I am able to confront people who worsen my body image.**  0 100  Not confident at all Moderately confident Very confident |
| --- |
| 1. **I am able to ask other people for help when I cannot improve my body image.**   0 100   Not confident at all Moderately confident Very confident |

**[INSERT ‘NEXT’ OR ‘>>’ BUTTON]**

**[PAGE BREAK]**

**[Present the following questions to adolescent in GROUP 1 only]:**

**[ACCEPTABILITY QUESTIONS - to be presented in BR P]**

**We are now going to ask you some questions about your experience of speaking with Dandara or Gabriel on Topity.**

**Using the scale, please indicate how you feel about each statement…**

- - - - 1. **How interesting was your conversation with Dandara or Gabriel (e.g., the information, tasks and storyline)?**

1 2 3 4 5 6 7

Not at all Very
Interesting Interesting

- - - - 1. **How much did you enjoy talking with Dandara or Gabriel?**

1 2 3 4 5 6 7

Not at all Very much

- - - - 1. **Did Dandara or Gabriel discuss topics that were important to you?**

1 2 3 4 5 6 7

Not at all Very much

**[INSERT ‘NEXT’ OR ‘>>’ BUTTON]**

**[PAGE BREAK]**

- - - - 1. **Do you think that Dandara or Gabriel discussed things that are important to other people your age?**

1 2 3 4 5 6 7

Not at all Very much

- - - - 1. **How comfortable did you feel talking about your body image with Dandara or Gabriel?**

1 2 3 4 5 6 7

Not at all Very

Comfortable Comfortable

- - - - 1. **How helpful was Topity at giving you skills to improve your body image?**

1 2 3 4 5 6 7

Not at all Very

Helpful Helpful

- - - - 1. **Would you recommend Topity to a friend, who is worried about their body image?**

1 2 3 4 5 6 7

I Definitely I Definitely

Would Not Would

**[INSERT ‘NEXT’ OR ‘>>’ BUTTON]**

**[PAGE BREAK]**

- - - - 1. **Would you like to use Topity again, in the future?**

1 2 3 4 5 6 7

I Definitely I Definitely

Would Not Would

- - - - 1. **How easy was Topity to use?**

1 2 3 4 5 6 7

Not At All Very Easy

Easy

- - - - 1. **How quickly did Topity address your questions or concerns?**

1 2 3 4 5 6 7

Very Slow Very Quick

- - - - 1. **How accurate was Topity’s response to your questions or concerns?**

1 2 3 4 5 6 7

Not at all Very Accurate

Accurate

**[INSERT ‘NEXT’ OR ‘>>’ BUTTON]**

**[PAGE BREAK]**

**[Present the following questions to adolescent in GROUP 1 only]**


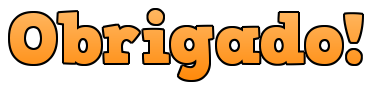


Thank you for providing your feedback in this survey. The information that you provide is very valuable.

If you found the chatbot helpful, we recommend that you **use the chatbot** **as often as you need or want**. Remember, the **more you engage** with the chatbot, the **better** your **confidence** and **self-esteem** is likely to be.

Please continue to the final page to receive instructions on the next phase of the project!

**[Present the following questions to adolescent in GROUP 2 only]**


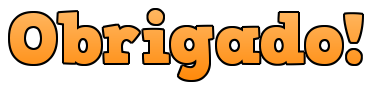


Thank you for providing your feedback in this survey. The information that you provide is very valuable. Please continue to the final page to receive instructions on the next phase of the project!

**[INSERT ‘NEXT’ OR ‘>>’ BUTTON]**

**[PAGE BREAK]**

**[All participants are to view the next page]**

**Next steps**

We will contact you again, in one week’s time, with a link to the next survey!

If you **do not** receive an email with the link in **seven days**, please contact **[insert Toluna contact].**

**Fontes de apoio para os jovens.**

Se você foi afetado por qualquer assunto levantado durante o preenchimento desse questionário, sugerimos inicialmente que você procure um familiar ou amigo de confiança para obter apoio ou discutir qualquer dúvida ou preocupação em relação ao seu bem-estar.

Se você sentir que isso não é possível ou se sentir mais confortável em procurar apoio em outro lugar, veja abaixo uma lista de serviços nacionais e locais que podem ser capazes de ajudar.

**Apoio Nacional**

Os seguintes links são boas fontes de apoio para qualquer pessoa procurando ajuda e conselhos confidenciais:

- **Transtornos Alimentares:**
  - ASTRAL – Associação Brasileira de Transtornos Alimentares: <https://astralbr.org/buscar-ajuda/>

**Apoio Local**

- **Transtornos Alimentares:**
  - AMBULIM: [http://www.ambulim.org.br](http://www.ambulim.org.br/) [São Paulo]
  - PROATA: [https://www.proata.com.br/](https://eur01.safelinks.protection.outlook.com/?url=https%3A%2F%2Fwww.proata.com.br%2F&data=02%7C01%7CHarriet5.Smith%40uwe.ac.uk%7Cc2d2059b611749600e1f08d81c32d810%7C07ef1208413c4b5e9cdd64ef305754f0%7C0%7C0%7C637290351984265808&sdata=RPgwjnOIhQ27Zq88cjG5XTzQGqB9MiWdWOmVjZrG4dI%3D&reserved=0) [São Paulo]

Thank you again for participating in our research!

**[End of survey 2]**

**[Start of Survey 3]**

**1 WEEK FOLLOW UP MEASURES**

(BODY IMAGE, MOOD, SELF-EFFICACY)

**SURVEY THREE**


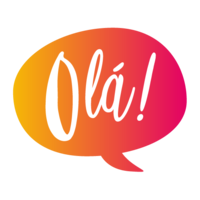


Welcome back! Thank you for all of your help so far. Today you will be completing your third survey, which should take approximately 15 minutes.

Please remember there are no right or wrong answers. Please complete the survey on your own, in private.

When you are ready to start, please press the NEXT button.

**[INSERT ‘NEXT’ OR ‘>>’ BUTTON]**

**[PAGE BREAK]**

**[Present the following questions to adolescent in GROUP 1 only]**

**[CHATBOT USAGE]**

**First, we would like to ask you about your chatbot usage over the past seven days.**

**Have you talked with Dandara and/or Gabriel in the past 7 days?**

- Sim
- Não

**[INSERT ‘NEXT’ OR ‘>>’ BUTTON]**

**[PAGE BREAK]**

**[Participants in both groups should be directed to this page, following the CHATBOT USAGE page]**

**[BODY ESTEEM MEASURE - to be presented in BR P]**

**Using the scale below, please mark how often each of the following statements applies to you.**

|  | Never  1 | Rarely  2 | Sometimes  3 | Often  4 | Always  5 |
| --- | --- | --- | --- | --- | --- |
| 1. I like how I look in photos. |  |  |  |  |  |
| 1. Other people consider me beautiful. |  |  |  |  |  |
| 1. I am proud of my body. |  |  |  |  |  |
| 1. I am preoccupied with trying to change my weight. |  |  |  |  |  |
| 1. I think my looks would help me to get a job. |  |  |  |  |  |
| [INSERT ‘NEXT’ OR ‘>>’ BUTTON]  [PAGE BREAK] | | | | |  |
| 1. I like what I see in the mirror. |  |  |  |  |  |
| 1. I would change lots of things about my appearance if I could. |  |  |  |  |  |
| 1. I am satisfied (happy) with my weight. |  |  |  |  |  |
| 1. I would like to be more beautiful. |  |  |  |  |  |
| 1. I really like how much I weigh. |  |  |  |  |  |
| 1. I would like to look like someone else. |  |  |  |  |  |
| [INSERT ‘NEXT’ OR ‘>>’ BUTTON]  [PAGE BREAK] | | | | |  |
| 1. People my own age like my appearance/the way I look. |  |  |  |  |  |
| 1. For this item, please select ‘Always’ |  |  |  |  |  |
| 1. My appearance makes me sad. |  |  |  |  |  |
| 1. I am as beautiful as most people. |  |  |  |  |  |
| 1. I am really happy with how I look/my appearance. |  |  |  |  |  |
| 1. I have the right weight for my height. |  |  |  |  |  |
| 1. I feel ashamed of my appearance. |  |  |  |  |  |
| [INSERT ‘NEXT’ OR ‘>>’ BUTTON]  [PAGE BREAK] | | | | | |
| 1. When I weight myself I get depressed (sad). |  |  |  |  |  |
| 1. My weight makes me unhappy. |  |  |  |  |  |
| 1. My appearance helps me get romantic dates. |  |  |  |  |  |
| 1. I get distressed about my appearance. |  |  |  |  |  |
| 1. I think I have a good body. |  |  |  |  |  |
| 1. I am as beautiful as I would like to be. |  |  |  |  |  |

**[INSERT ‘NEXT’ OR ‘>>’ BUTTON]**

**[PAGE BREAK]**

**[FILLER ITEMS - to be presented in BR P]**

**Using the scale, please indicate how you feel about each statement…**

- **Spending time with my friends is important to me.**

1 2 3 4 5 6 7 8 9 10
Not at all Very much

- **I enjoy being physically active.**

1 2 3 4 5 6 7 8 9 10
Not at all Very much

- **Having a good sleep routine is important to me.**

1 2 3 4 5 6 7 8 9 10
Not at all Very much

**[INSERT ‘NEXT’ OR ‘>>’ BUTTON]**

**[PAGE BREAK]**

**[MOOD MEASURE - to be presented in BR P]**

**Please indicate how much you felt this way in the past week…**

|  | Not even a little  1 | A little  2 | So-so  3 | Quite  4 | Very much  5 |
| --- | --- | --- | --- | --- | --- |
| Divertido (fun) |  |  |  |  |  |
| Alegre (joyful) |  |  |  |  |  |
| Contente (content) |  |  |  |  |  |
| Animado (cheerful) |  |  |  |  |  |
| Incomodado (bothered) |  |  |  |  |  |
| Humilhado (humiliated) |  |  |  |  |  |
| Magoado (hurt) |  |  |  |  |  |
| Irritado (irritated) |  |  |  |  |  |

**[INSERT ‘NEXT’ OR ‘>>’ BUTTON]**

**[PAGE BREAK]**

**[FILLER ITEMS]**

**Using the scale, please indicate how you feel about each statement…**

- **Doing well at school is important to me.**

1 2 3 4 5 6 7 8 9 10
Not at all Very much

- **Please select the number 8.**

1 2 3 4 5 6 7 8 9 10

Not at all Very much

- **I enjoy trying new things.**

1 2 3 4 5 6 7 8 9 10
Not at all Very much

- **Getting along with other people is important to me.**

1 2 3 4 5 6 7 8 9 10
Not at all Very much

**[INSERT ‘NEXT’ OR ‘>>’ BUTTON]**

**[PAGE BREAK]**

**[BODY IMAGE SELF-EFFICACY MEASURE - to be presented in BR P]**

*The term ‘body image’ takes into account your thoughts, feelings and behaviours in relation to your body. That is, it does not only refer to its appearance. Takes this into account when answering the scale below.*

Please rate your level of confidence for each item on the scale from 0-100

| 1. **I am able to learn and practice new skills to help improve my body image**   0 100  Not confident at all Moderately confident Very confident |
| --- |
| 1. **I am able to recognize situations that will worsen my body image***.*   0 100  Not confident at all Moderately confident Very confident |
| 1. **I am able to perform daily activities that improve my body image.**  0 100  Not confident at all Moderately confident Very confident |

**[INSERT ‘NEXT’ OR ‘>>’ BUTTON]**

**[PAGE BREAK]**

| 1. **I am able to confront people who worsen my body image.**  0 100  Not confident at all Moderately confident Very confident |
| --- |
| 1. **I am able to ask other people for help when I cannot improve my body image.**   0 100   Not confident at all Moderately confident Very confident |

**[INSERT ‘NEXT’ OR ‘>>’ BUTTON]**

**[PAGE BREAK]**

**[Present the following questions to adolescent in GROUP 1 only]**


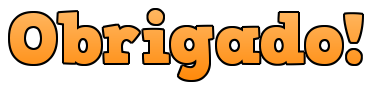


Thank you for providing your feedback in this survey. The information that you provide is very valuable.

If you found the chatbot helpful, we **recommend that use the chatbot** **as often as you need or want**. Remember, the **more you engage** with the chatbot, the **better** your **confidence** and **self-esteem** is likely to be.

Please continue to the final page to receive instructions on the next phase of the project!

**[Present the following questions to adolescent in GROUP 2 only]**


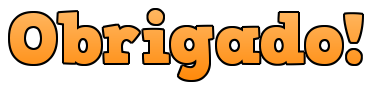


Thank you for providing your feedback in this survey. The information that you provide is very valuable. Please continue to the final page to receive instructions on the next phase of the project!

**[INSERT ‘NEXT’ OR ‘>>’ BUTTON]**

**[PAGE BREAK]**

**[All participants are to view the next page]**

**[Present the list of resources]**

**Next steps**

We will contact you again, in three weeks’ time, with a link to the next survey!

If you **do not** receive an email with the link in **three weeks**, please contact **[insert Toluna contact].**

**Fontes de apoio para os jovens.**

Se você foi afetado por qualquer assunto levantado durante o preenchimento desse questionário, sugerimos inicialmente que você procure um familiar ou amigo de confiança para obter apoio ou discutir qualquer dúvida ou preocupação em relação ao seu bem-estar.

Se você sentir que isso não é possível ou se sentir mais confortável em procurar apoio em outro lugar, veja abaixo uma lista de serviços nacionais e locais que podem ser capazes de ajudar.

**Apoio Nacional**

Os seguintes links são boas fontes de apoio para qualquer pessoa procurando ajuda e conselhos confidenciais:

- **Transtornos Alimentares:**
  - ASTRAL – Associação Brasileira de Transtornos Alimentares: <https://astralbr.org/buscar-ajuda/>

**Apoio Local**

- **Transtornos Alimentares:**
  - AMBULIM: [http://www.ambulim.org.br](http://www.ambulim.org.br/) [São Paulo]
  - PROATA: [https://www.proata.com.br/](https://eur01.safelinks.protection.outlook.com/?url=https%3A%2F%2Fwww.proata.com.br%2F&data=02%7C01%7CHarriet5.Smith%40uwe.ac.uk%7Cc2d2059b611749600e1f08d81c32d810%7C07ef1208413c4b5e9cdd64ef305754f0%7C0%7C0%7C637290351984265808&sdata=RPgwjnOIhQ27Zq88cjG5XTzQGqB9MiWdWOmVjZrG4dI%3D&reserved=0) [São Paulo]

**[End of survey 3]**

**[Start of Survey 4]**

**1 MONTH FOLLOW UP MEASURES**

(BODY IMAGE, MOOD, SELF-EFFICACY)

**SURVEY FOUR**


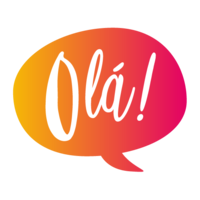


Welcome back! Thank you for all of your help so far. Today you will be completing your fourth survey, which should take approximately 15 minutes.

Please remember that there are no right or wrong answers. Please complete the survey on your own, in private.

Continue to the next page, when you are ready to get started!

**[INSERT ‘NEXT’ OR ‘>>’ BUTTON]**

**[PAGE BREAK]**

**[Present the following questions to adolescent in GROUP 1 only]**

**[CHATBOT USAGE]**

**First, we would like to ask you about your chatbot usage over the past three weeks.**

**Have you talked with Dandara and/or Gabriel in the past three weeks?**

- Sim (yes)
- Não (no)

**[INSERT ‘NEXT’ OR ‘>>’ BUTTON]**

**[PAGE BREAK]**

**[Participants in both groups should be directed to this page, following the CHATBOT USAGE page]**

**[BODY ESTEEM MEASURE – to be presented in BR P]**

**Using the scale below, please mark how often each of the following statements applies to you.**

|  | Never  1 | Rarely  2 | Sometimes  3 | Often  4 | Always  5 |
| --- | --- | --- | --- | --- | --- |
| 1. I like how I look in photos. |  |  |  |  |  |
| 1. Other people consider me beautiful. |  |  |  |  |  |
| 1. I am proud of my body. |  |  |  |  |  |
| 1. I am preoccupied with trying to change my weight. |  |  |  |  |  |
| 1. I think my looks would help me to get a job. |  |  |  |  |  |
| [INSERT ‘NEXT’ OR ‘>>’ BUTTON]  [PAGE BREAK] | | | | |  |
| 1. I like what I see in the mirror. |  |  |  |  |  |
| 1. I would change lots of things about my appearance if I could. |  |  |  |  |  |
| 1. I am satisfied (happy) with my weight. |  |  |  |  |  |
| 1. I would like to be more beautiful. |  |  |  |  |  |
| 1. I really like how much I weigh. |  |  |  |  |  |
| 1. I would like to look like someone else. |  |  |  |  |  |
| [INSERT ‘NEXT’ OR ‘>>’ BUTTON]  [PAGE BREAK] | | | | |  |
| 1. People my own age like my appearance/the way I look. |  |  |  |  |  |
| 1. For this item, please select ‘1’ |  |  |  |  |  |
| 1. My appearance makes me sad. |  |  |  |  |  |
| 1. I am as beautiful as most people. |  |  |  |  |  |
| 1. I am really happy with how I look/my appearance. |  |  |  |  |  |
| 1. I have the right weight for my height. |  |  |  |  |  |
| 1. I feel ashamed of my appearance. |  |  |  |  |  |
| [INSERT ‘NEXT’ OR ‘>>’ BUTTON]  [PAGE BREAK] | | | | | |
| 1. When I weight myself I get depressed (sad). |  |  |  |  |  |
| 1. My weight makes me unhappy. |  |  |  |  |  |
| 1. My appearance helps me get romantic dates. |  |  |  |  |  |
| 1. I get distressed about my appearance. |  |  |  |  |  |
| 1. I think I have a good body. |  |  |  |  |  |
| 1. I am as beautiful as I would like to be. |  |  |  |  |  |

**[INSERT ‘NEXT’ OR ‘>>’ BUTTON]**

**[PAGE BREAK]**

**[FILLER ITEMS – to be presented in BR P]**

**Using the scale, please indicate how you feel about each statement…**

- **Spending time with my friends is important to me.**

1 2 3 4 5 6 7 8 9 10
Not at all Very much

- **I enjoy being physically active.**

1 2 3 4 5 6 7 8 9 10
Not at all Very much

- **Having a good sleep routine is important to me.**

1 2 3 4 5 6 7 8 9 10
Not at all Very much

**[INSERT ‘NEXT’ OR ‘>>’ BUTTON]**

**[PAGE BREAK]**

**[MOOD MEASURE – to be presented in BR P]**

**Please indicate how much you felt this way in the past week…**

|  | Not even a little  1 | A little  2 | So-so  3 | Quite  4 | Very much  5 |
| --- | --- | --- | --- | --- | --- |
| Divertido (fun) |  |  |  |  |  |
| Alegre (joyful) |  |  |  |  |  |
| Contente (content) |  |  |  |  |  |
| Animado (cheerful) |  |  |  |  |  |
| Incomodado (bothered) |  |  |  |  |  |
| Humilhado (humiliated) |  |  |  |  |  |
| Magoado (hurt) |  |  |  |  |  |
| Irritado (irritated) |  |  |  |  |  |

**[INSERT ‘NEXT’ OR ‘>>’ BUTTON]**

**[PAGE BREAK]**

**[FILLER ITEMS - to be presented in BR P]**

**Using the scale, please indicate how you feel about each statement…**

- **Doing well at school is important to me.**

1 2 3 4 5 6 7 8 9 10
Not at all Very much

- **Please select the number 8.**

1 2 3 4 5 6 7 8 9 10

Not at all Very much

- **I enjoy trying new things.**

1 2 3 4 5 6 7 8 9 10
Not at all Very much

- **Getting along with other people is important to me.**

1 2 3 4 5 6 7 8 9 10
Not at all Very much

**[INSERT ‘NEXT’ OR ‘>>’ BUTTON]**

**[PAGE BREAK]**

**[BODY IMAGE SELF-EFFICACY MEASURE - to be presented in BR P]**

*The term ‘body image’ takes into account your thoughts, feelings and behaviours in relation to your body. That is, it does not only refer to its appearance. Takes this into account when answering the scale below.*

Please rate your level of confidence for each item on the scale from 0-100

| 1. **I am able to learn and practice new skills to help improve my body image**   0 100  Not confident at all Moderately confident Very confident |
| --- |
| 1. **I am able to recognize situations that will worsen my body image***.*   0 100  Not confident at all Moderately confident Very confident |
| 1. **I am able to perform daily activities that improve my body image.**  0 100  Not confident at all Moderately confident Very confident |

**[INSERT ‘NEXT’ OR ‘>>’ BUTTON]**

**[PAGE BREAK]**

| 1. **I am able to confront people who worsen my body image.**  0 100  Not confident at all Moderately confident Very confident |
| --- |
| 1. **I am able to ask other people for help when I cannot improve my body image.**   0 100   Not confident at all Moderately confident Very confident |

**[INSERT ‘NEXT’ OR ‘>>’ BUTTON]**

**[PAGE BREAK]**

**[Present the below information to GROUP 1 only]**


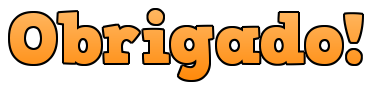


Congratulations, you have now completed all of the questions in the survey!

You have also come to the end of this research project. Thank you for your help, your participation and feedback are extremely valuable.

**Next Steps**

You will be reimbursed for your internet usage, depending on which group you participated in. A member from the research agency, Toluna, will contact you via email with more details.

**Fontes de apoio para os jovens.**

Se você foi afetado por qualquer assunto levantado durante o preenchimento desse questionário, sugerimos inicialmente que você procure um familiar ou amigo de confiança para obter apoio ou discutir qualquer dúvida ou preocupação em relação ao seu bem-estar.

Se você sentir que isso não é possível ou se sentir mais confortável em procurar apoio em outro lugar, veja abaixo uma lista de serviços nacionais e locais que podem ser capazes de ajudar.

**Apoio Nacional**

Os seguintes links são boas fontes de apoio para qualquer pessoa procurando ajuda e conselhos confidenciais:

- **Transtornos Alimentares:**
  - ASTRAL – Associação Brasileira de Transtornos Alimentares: <https://astralbr.org/buscar-ajuda/>

**Apoio Local**

- **Transtornos Alimentares:**
  - AMBULIM: [http://www.ambulim.org.br](http://www.ambulim.org.br/) [São Paulo]
  - PROATA: [https://www.proata.com.br/](https://eur01.safelinks.protection.outlook.com/?url=https%3A%2F%2Fwww.proata.com.br%2F&data=02%7C01%7CHarriet5.Smith%40uwe.ac.uk%7Cc2d2059b611749600e1f08d81c32d810%7C07ef1208413c4b5e9cdd64ef305754f0%7C0%7C0%7C637290351984265808&sdata=RPgwjnOIhQ27Zq88cjG5XTzQGqB9MiWdWOmVjZrG4dI%3D&reserved=0) [São Paulo]

Thank you again for participating in our research!

**[INSERT ‘NEXT’ OR ‘>>’ BUTTON]**

**[PAGE BREAK]**

**[Present the below information to GROUP 2 only]**


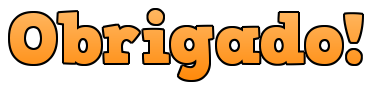


Congratulations, you have now completed all of the questions in the survey! You have also come to the end of this research project. Thank you for your help, your participation and feedback are extremely valuable.

**Next Steps**

You will be reimbursed for your time and internet usage, depending on which group you participated in. A member from the research agency, Toluna, will contact you via email with more details.

Over the next week, our research team will send you a link to the chatbot. You do not have to engage with the chatbot, but it will be available for you to experience and engage with. You will not be compensated for your engagement with the chatbot.

Many young people in Brazil report feeling sad or unhappy about how they look. Our chatbot uses special technology to speak with young people about their body image concerns. In the chatbot, users are able choose between two chatbot characters to engage with, either Dandara or Gabriel. Dandara and Gabriel are not real people; they are robots that have been carefully designed to help young people discuss their worries. Dandara and/or Gabriel provide users with tools and strategies that may help those with body image concerns.

**Fontes de apoio para os jovens.**

Se você foi afetado por qualquer assunto levantado durante o preenchimento desse questionário, sugerimos inicialmente que você procure um familiar ou amigo de confiança para obter apoio ou discutir qualquer dúvida ou preocupação em relação ao seu bem-estar.

Se você sentir que isso não é possível ou se sentir mais confortável em procurar apoio em outro lugar, veja abaixo uma lista de serviços nacionais e locais que podem ser capazes de ajudar.

**Apoio Nacional**

Os seguintes links são boas fontes de apoio para qualquer pessoa procurando ajuda e conselhos confidenciais:

- **Transtornos Alimentares:**
  - ASTRAL – Associação Brasileira de Transtornos Alimentares: <https://astralbr.org/buscar-ajuda/>

**Apoio Local**

- **Transtornos Alimentares:**
  - AMBULIM: [http://www.ambulim.org.br](http://www.ambulim.org.br/) [São Paulo]
  - PROATA: [https://www.proata.com.br/](https://eur01.safelinks.protection.outlook.com/?url=https%3A%2F%2Fwww.proata.com.br%2F&data=02%7C01%7CHarriet5.Smith%40uwe.ac.uk%7Cc2d2059b611749600e1f08d81c32d810%7C07ef1208413c4b5e9cdd64ef305754f0%7C0%7C0%7C637290351984265808&sdata=RPgwjnOIhQ27Zq88cjG5XTzQGqB9MiWdWOmVjZrG4dI%3D&reserved=0) [São Paulo]

Thank you again for participating in our research!

**[INSERT ‘NEXT’ OR ‘>>’ BUTTON]**

**[PAGE BREAK]**

**[End of survey 4]**

**[End of the project]**


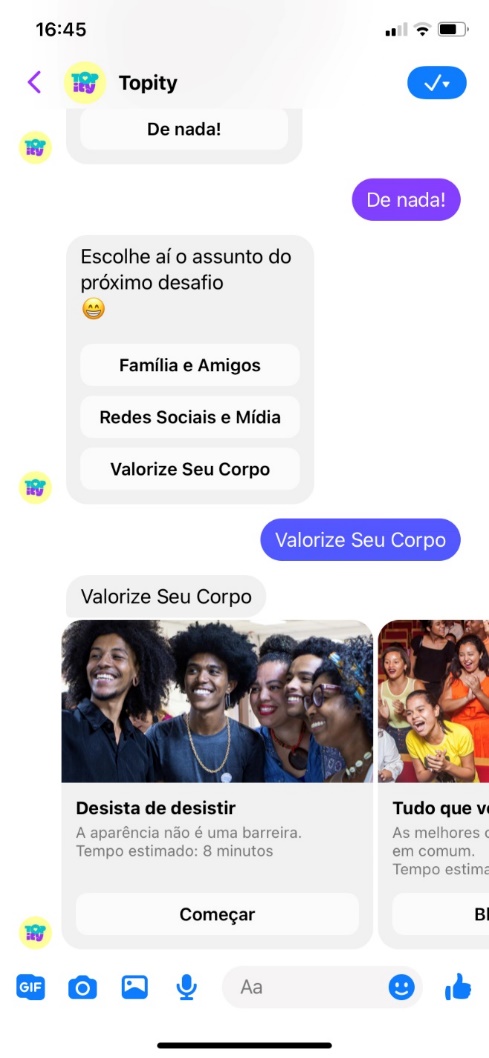

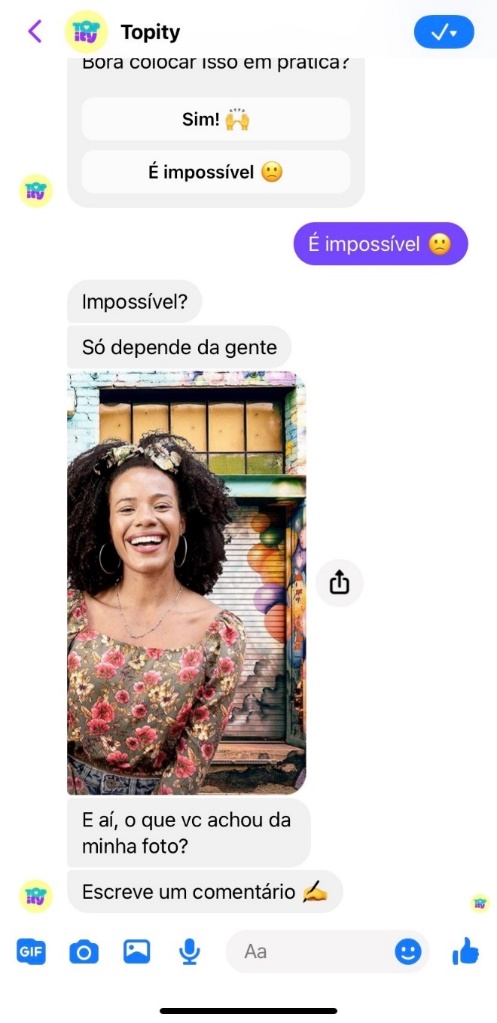

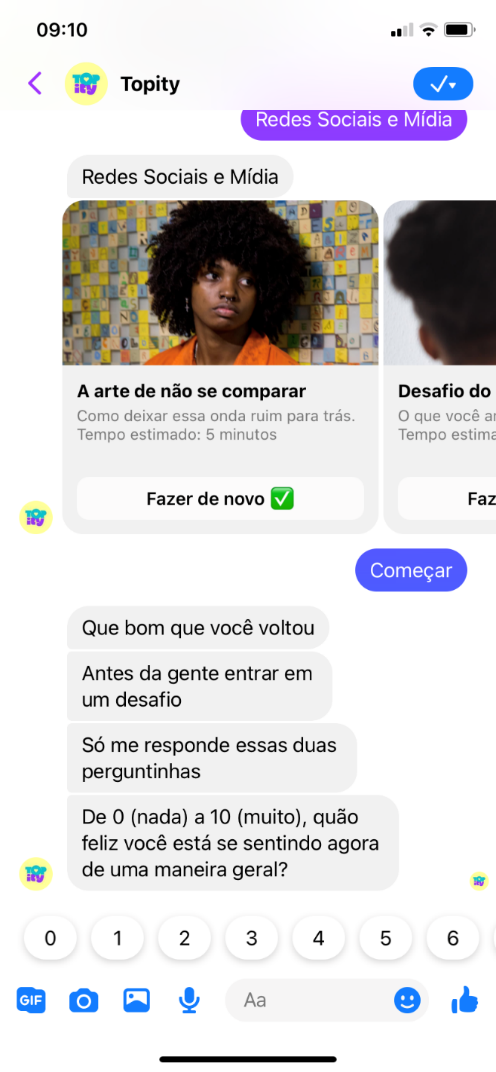


*Supplementary Fig 1.* Screenshots of a Topity conversation on mobile view.

| Supplementary Table 1. Acceptability scores by age and gender. | | | | | | | | | |
| --- | --- | --- | --- | --- | --- | --- | --- | --- | --- |
|  | *Girls* | | |  | *Boys* | | | *Whole Sample* | |
|  | *13 – 15 years old (M, SD)* | *16 – 18 years old*  *(M, SD)* |  | | | *13 – 15 years old (M, SD)* | *16 – 18 years old (M, SD)* | | *13 – 18 years old (M, SD)* |
| *Acceptability Items* |  |  |  | | |  |  | |  |
| Emotive Response | 6.65 (0.37) | 6. 32 (0.99) |  | | | 6.25 (0.98) | 5.63 (1.44) | | 6.25 (1.05) |
| 1. How interesting was your conversation with Dandara and/or Gabriel (e.g., the information, tasks and storyline)? | 6.66 (0.58) | 6.45 (0.92) |  | | | 6.29 (1.25) | 5.58 (1.85) | | 6.29 (1.25) |
| 2. How much did you enjoy talking with Dandara and/or Gabriel? | 6.86 (0.34) | 6.50 (0.98) |  | | | 6.15 (1.35) | 5.53 (1.83) | | 6.32 (1.27) |
| 3. How comfortable did you feel talking about your body image with Dandara and/or Gabriel? | 6.42 (0.73) | 6.02 (1.46) |  | | | 6.26 (1.02) | 5.84 (1.31) | | 6.15 (1.20) |
| Relevance | 6.52 (0.52) | 6.45 (0.77) |  | | | 6.35 (0.79) | 5.82 (1.35) | | 6.33 (0.92) |
| 4. Did the chatbot discuss things that were important to you? | 6.59 (0.83) | 6.68 (0.55) |  | | | 6.48 (0.8) | 6.06 (1.52) | | 6.49 (0.97) |
| 5. Did the chatbot discuss things that are important to other young people your age? | 6.72 (0.56) | 6.72 (0.82) |  | | | 6.66 (0.78) | 6.39 (1.10) | | 6.65 (0.82) |
| 6. How helpful was the chatbot at giving you skills to improve your body image? | 6.22 (0.98) | 5.95 (1.30) |  | | | 5.89 (1.42) | 5.10 (2.04) | | 5.86 (1.48) |
| Ease of Use | 6.24 (0.93) | 6.13 (0.91) |  | | | 6.23 (0.78) | 6.12 (0.91) | | 6.19 (0.88) |
| 7. How quickly did the chatbot address your questions or concerns? 8. How easy was the chatbot to use? 9. How accurate was the chatbot’s response to your questions or concerns? | 6.10 (1.2)  6.50 (0.94)  6.10 (1.2) | 6.11 (1.08)  6.30 (1.3)  5.98 (1.13) |  | | | 6.19 (1.24)  6.41 (1.37)  6.11 (1.15) | 6.23 (0.89)  6.40 (1.22)  5.73 (1.55) | | 6.15 (1.09)  6.41 (1.18)  6.0 (1.24) |
| Likelihood to Recommend/Re-engage | 6.63 (0.58) | 6.28 (1.13) |  | | | 6.04 (1.14) | 5.62 (1.63) | | 6.20 (1.19) |
| 10. Would you recommend this chatbot to a friend, who is worried about their body image? 11. Would you talk to this chatbot again, in the future? | 6.75 (0.60)  6.53 (0.91) | 6.50 (0.95)  6.08 (1.46) |  | | | 6.55 (1.01)  5.52 (1.74) | 6.06 (1.55)  5.17 (2.00) | | 6.49 (1.06)  5.92 (1.58) |
| Overall Acceptability (11 items) | 6.51 (0.45) | 6.30 (0.77) |  | | | 6.25 (0.75) | 5.74 (1.18) | | 6.23 (0.85) |
